# Intestinal lipase inhibition uncouples diet-induced obesity from MASH by remodeling the hepatic lipidome in mice

**DOI:** 10.1016/j.jlr.2026.101112

**Published:** 2026-07-27

**Authors:** Alvin P. Chan, Tomasz J. Czernuszewicz, Rochelle W. Lai, Kelsey E. Jarrett, Andrew Lau, Anisa Warshan, Angela S. Cheng, Michelle E. Steel, Heidi M. Schmidt, Jakeline Larios, Isaiah Little, Gabriella E. Rubert, Madelaine C. Brearley-Sholto, Zeinab Mamouei, Jessica K. Pesner, Christopher D. Hamad, Kevin P. Francis, Nicholas M. Bernthal, Elizabeth F. Redente, Clara E. Magyar, Samual W. French, Kevin J. Wiliams, Elizabeth J. Tarling, Thomas Q. de Aguiar Vallim

**Affiliations:** 1 Division of Cardiology, Department of Medicine, David Geffen School of Medicine, University of California, Los Angeles (UCLA), Los Angeles, CA, USA; 2 Division of Gastroenterology, Hepatology and Nutrition, Department of Pediatrics, Mattel Children's Hospital, David Geffen School of Medicine, University of California, Los Angeles (UCLA), Los Angeles, CA, USA; 3 Revvity, Inc., Waltham, MA, USA; 4 Department of Biological Chemistry, David Geffen School of Medicine, University of California, Los Angeles (UCLA), Los Angeles, CA, USA; 5 Molecular Biology Institute, University of California, Los Angeles (UCLA), Los Angeles, CA, USA; 6 Department of Orthopedic Surgery, David Geffen School of Medicine, University of California, Los Angeles (UCLA), Los Angeles, CA, USA; 7 Department of Pediatrics, National Jewish Health, Denver, CO, USA; 8 Translational Pathology Core Laboratory, University of California, Los Angeles (UCLA), Los Angeles, CA, USA; 9 Department of Pathology and Laboratory Medicine, David Geffen School of Medicine, University of California, Los Angeles (UCLA), Los Angeles, CA, USA; 10 UCLA Lipidomics Core Laboratory, University of California, Los Angeles (UCLA), Los Angeles, CA, USA; 11 Division of Endocrinology, Department of Medicine, David Geffen School of Medicine, University of California, Los Angeles (UCLA), Los Angeles, CA, USA; 12 Jonsson Comprehensive Cancer Center (JCCC), University of California, Los Angeles (UCLA), Los Angeles, CA, USA

**Keywords:** dietary fat, polyunsaturated fatty acid, lipase, digestive, cholesterol, absorption, liver, lipidomics, ultrasound, shear wave elastography

## Abstract

Dietary fatty acids exert distinct effects on hepatic metabolism. Intestinal lipase inhibition, used clinically for obesity, promotes weight loss by reducing dietary fat absorption, yet its impact on hepatic lipid metabolism in metabolic dysfunction-associated steatohepatitis (MASH) remains poorly defined. Here, we examined the metabolic consequences of lipase inhibition on fatty acid absorption in MASH. To induce MASH, C57BL/6J mice were fed the high-fat Gubra-Amylin NASH diet with or without the lipase inhibitor orlistat for 32 weeks. Orlistat protected against diet-induced obesity by promoting fat malabsorption, but failed to prevent the progression of hepatomegaly, steatosis, and fibrosis across longitudinal ultrasound imaging, biochemical assessments, and endpoint histology. Despite limiting weight gain, orlistat unexpectedly exacerbated hepatic inflammation. Lipidomic analyses revealed that the indiscriminate inhibition of fatty acid absorption by orlistat depleted the liver of essential PUFAs while increasing saturated fatty acids. This shift was associated with increased hepatic lipogenesis and proinflammatory signaling, preventing protection from MASH. In summary, lipase inhibition of intestinal fat absorption uncouples obesity from liver disease progression by reshaping the hepatic lipidome. These findings underscore the importance of dietary fat and hepatic lipid composition as key drivers of MASH pathogenesis.

Metabolic dysfunction-associated steatotic liver disease (MASLD) has emerged as a global epidemic, with prevalence rising in parallel with the worldwide surge in obesity. (1, 2, 3, 4) MASLD encompasses a spectrum of liver diseases, beginning with simple steatosis and progressing to metabolic dysfunction-associated steatohepatitis (MASH) with the development of inflammation, fibrosis, and ultimately cirrhosis. A hallmark of this disease is the excessive accumulation of hepatic lipids, driven by an imbalance between lipid input and lipid disposal. (5) In modern societies, where energy-dense diets are ubiquitous, excess intake leads to enhanced intestinal nutrient absorption, driving lipid accumulation in the liver, adipose tissue, and other metabolic organs. (6) As a result, MASLD and obesity frequently develop in parallel (4).

Accumulating evidence suggests that the types of dietary fatty acids, not merely their total amount, play a critical role in MASLD progression. (7) Lipidomic studies of MASLD/MASH consistently demonstrate an imbalance in hepatic fatty acid composition, with enrichment of saturated fatty acids and depletion of PUFAs. (8, 9) Saturated fatty acids exacerbate oxidative stress and inflammation, (10, 11) whereas PUFAs promote membrane fluidity, suppress de novo lipogenesis, and generate pro-resolving lipid mediators that attenuate inflammatory signaling pathways. (12, 13) Accordingly, current dietary strategies for both MASLD and obesity recommend limiting saturated fat intake and increasing dietary PUFAs. (14, 15, 16) We have recently demonstrated that the intestine, as the natural gatekeeper for fat entry into the body, is inherently primed to selectively absorb PUFAs over saturated fatty acids due to their greater solubility in mixed bile acid micelles, (17) a phenomenon that likely reflects evolutionarily conserved mechanisms to ensure adequate uptake of essential PUFAs during periods of food scarcity. Given this intrinsic selectivity in PUFA uptake, interventions that broadly block absorption of all types of dietary fatty acids may disrupt the physiologic balance of fatty acid species in the liver.

Orlistat, one of the first anti-obesity drugs approved in the United States in 1999, inhibits gastrointestinal lipases within the intestinal lumen and blocks triglyceride hydrolysis. (18, 19) By limiting the release of free fatty acids and monoacylglycerides from dietary triglycerides, orlistat reduces the absorption of dietary fat. When combined with physical activity or a low-fat diet, orlistat reduces body weight by decreasing fat absorption. (20, 21) In recent work, we showed that orlistat reduces the absorption of all fatty acids, abolishing any preferential uptake of PUFAs. (17) Thus, while orlistat lowers overall fat absorption, it remains unclear how its indiscriminate inhibition of fatty acid uptake influences the trajectory of MASH progression.

In this study, we investigated the impact of intestinal lipase inhibition in a dietary MASH model using the Gubra-Amylin NASH (GAN) diet. We utilized ultrasound imaging and shear wave elastography (SWE) to longitudinally and noninvasively track the development of hepatomegaly, steatosis, and fibrosis in mice over time. Using mass spectrometry-based lipidomics and fatty acid profiling, we characterized how changes in fatty acid absorption by orlistat reshapes the hepatic lipidome during MASH development. Our findings show that intestinal lipase inhibition uncouples obesity from MASH by remodeling hepatic lipidome, underscoring the critical role of hepatic fatty acid composition in disease progression.

## Materials and methods

### Animals

C57BL/6J male mice (000664) were obtained from The Jackson Laboratory at six weeks of age and acclimated at UCLA until experiment start. All mice were group-housed and maintained in standard cages under temperature-controlled conditions (20–24°C) with a 12 h:12 h light:dark cycle (dark phase, 18:00-06:00) in the vivarium. At eight weeks of age, mice were fed GAN diet (Research Diets, D09100310, 40% kcal fat, 20% kcal fructose, 2% cholesterol, 4.5 kcal/g) for 32 weeks to induce MASH. Mice in the orlistat group received orlistat via a modified GAN diet containing orlistat (TCI America, O0381) at 0.01% by weight (Research Diets, D22041402). Based on the average daily food intake in adult mice, this corresponds to an orlistat dosage of approximately 10 mg/kg/day. (17, 22) Body weight was measured weekly, and body composition (whole body fat and lean mass) was assessed by EchoMRI™ (Houston, TX) approximately every eight weeks. Plasma was collected via retro-orbital survival bleeds following a four hour fast approximately every eight weeks. Food consumption per cage was measured over a 1-week period at weeks 8 and 16 and averaged across the number of mice to estimate mean daily food intake per mouse. Another cohort of mice was dedicated to serial ultrasound imaging over 32 weeks. Independent cohorts of control and orlistat-treated mice were used to measure de novo *lipogenesis* at week 8 and, in a separate cohort, at week 32. For tissue collection, mice were fasted for four hours prior to sacrifice. Plasma was collected under anesthesia via retro-orbital collection, and livers were collected and snap-frozen in liquid nitrogen. Tissues were stored at −80°C. All experimental protocols were approved by the UCLA Animal Research Committee.

### In vivo ultrasound imaging

Imaging was performed using a Vega™ automated ultrasound imager (Revvity, Inc., Durham, NC). Mice were fasted for four hours and anesthetized with vaporized isoflurane (3% induction and 1.5% maintenance) for imaging. The abdomen was shaved and depilated with Nair, and mice were positioned prone on the acoustic membrane with a thin layer of water for coupling. Body temperature was maintained with an overhead heat lamp. For each mouse, a wide-field 3D B-mode scout scan was acquired for liver localization and volume measurement (24 MHz, 2 focal zones at 7 and 16 mm with 30 mm depth setting, lateral × elevational ROI dimensions ∼50 × 35 mm, 0.1 mm elevation step size), followed by a 3D SWE scan for liver stiffness and echogenicity measurements (lateral × elevational ROI dimensions ∼20 × 5 mm, 2 mm frame step size). To quantify liver volume, wide-field scout scans were passed through a custom artificial intelligence segmentation model (Living Image™ Synergy AI software, Revvity, Inc., Waltham, MA) developed and trained specifically for Vega liver volumetry. The artificial intelligence model consisted of a modified U-Net (23) implemented in PyTorch and MONAI framework (24) and trained on an NVIDIA RTX 3090 GPU. The model, which was developed prior to this study, was trained on n = 522 Vega images segmented by an expert human reader using a conventional 80-20 split for training and validation. Following inference, the artificial intelligence-generated segmentations were then loaded in SonoEQ v1.14.3 (Revvity, Inc.) and verified by an expert human reader for accuracy. To quantify liver stiffness and echogenicity, paired B-mode and SWE volumes were segmented manually in SonoEQ using the Draw effect to outline the liver contour in each axial slice of the 3D volume. Finally, the computed values for median echogenicity (in a.u.) and median stiffness (in kPa, Young’s modulus) were exported to CSV files for further statistical analysis and plotting. SWE confidence threshold was set to 0.75 and minimum stiffness threshold was set to 0.75 kPa.

### Cholesterol and fatty acid analysis

Feces, livers, and diet were also analyzed for cholesterol and fatty acid content. Cholesterol and fatty acid methyl esters were extracted from feces, liver, and diet samples and quantified by GC-MS, as described previously (17, 25).

### In vivo hepatic lipogenesis and cholesterol synthesis

To assess hepatic lipogenesis and cholesterol synthesis, drinking water was supplemented with deuterium (Sigma-Aldrich, 151882) at 5% concentration during the final three days of the experiments. Lipid synthesis was quantified through GC-MS analysis of deuterium-labeled isotopic enrichment in fatty acid and cholesterol methyl esters, as described previously (17, 26).

### Lipidomic analysis

Liver tissues were prepared and analyzed on the Sciex 5500 with DMS device (Lipidyzer Platform) with an expanded targeted acquisition list consisting of 1450 lipid species across 17 subclasses, as previously described. (17, 27) Quantitative values were normalized to mg of tissue.

### Absorption studies

All feces were collected at week 32 for quantification of total fecal mass, fecal energy loss, and fecal fatty acid excretion. Fecal energy loss was determined by measuring the heat of combustion of fecal samples using a 6400 isoperibol calorimeter equipped with an 1138 oxygen bomb (Parr Instruments Co., Moline, IL). Fecal fatty acid and cholesterol excretion was measured by GC-MS analysis of fecal samples. Total fecal energy loss (kcal/week), fecal fatty acid loss (μmol/week), and fecal cholesterol loss (nmol/week) were calculated by multiplying cumulative fecal mass (g/week) by fecal energy density (kcal/g), fecal fatty acid content (μmol/g), and fecal cholesterol content (nmol/g), respectively.

Absorption studies were performed by incorporating sucrose polybehenate (SPB) into the diet as a nonabsorbable tracer. (28) To measure absorption at weeks 16 and 32, mice were fed either GAN diet containing 1.5% SPB (Research Diets, D22041401) or GAN diet containing 0.01% orlistat and 1.5% SPB (Research Diets, D23013101) for 24 h before returning to their original diet. Feces were collected over the subsequent 24 h. Fatty acid absorption was determined from the ratio of behenic acid to other fatty acids in diet and feces by GC-MS analysis of fatty acid methyl esters. Fatty acid absorption was expressed as fractional absorption (%).

### Liver function tests

Plasma ALT and AST were measured using Teco Diagnostics ALT and AST Liquid Reagent Kinetic Method Kits (A524150 and A559150) according to manufacturer instructions, as previously described (17).

### Glucose tolerance tests

In a separate 8-week experiment, oral glucose tolerance tests were performed in control and orlistat-treated mice after seven weeks of GAN diet feeding. Mice were fasted for four hours and administered D-glucose (Sigma-Aldrich, G8769) by oral gavage dosed at 1.5 g/kg body weight. Blood glucose was measured from tail vein blood at 0, 15, 30, 45, 60, 90, and 120 min after gavage using a OneTouch Ultra Mini glucometer (LifeScan, Milpitas, CA).

### Histological analysis

Liver tissues were collected and fixed in 4% paraformaldehyde for 24 h. Following fixation, samples were stored in 70% ethanol until processing, paraffin embedding, sectioning, and H&E staining by the UCLA Translational Pathology Core Laboratory, with picrosirius red staining performed by the Elizabeth Redente Lab at National Jewish Health in Denver, CO. Blinded histopathological assessment of H&E-stained slides was conducted by an expert liver pathologist using the MASLD Activity Score. The MASLD Activity Score represents a composite score of overall disease severity (0–8) derived from steatosis (0–3), lobular inflammation (0–3), and hepatocellular ballooning (0–2). (29) Hepatic fibrosis was quantified on picrosirius red-stained sections through digital image analysis of collagen proportionate area using HALO software (Indica Labs, Albuquerque, NM). Fibrosis severity was categorized as mild (<5% collagen proportionate area), moderate (5–10%), advanced (10–20%), or severe (>20%). (30) Brightfield images were obtained using a KEYENCE BZ-X810 Fluorescence Microscope (El Segundo, CA) at 200 × total magnification.

### Quantitative PCR analysis

RNA was extracted from snap frozen liver and analyzed for gene expression, as previously described, (17) using specific primers (Supplemental Table S1). All genes were normalized to housekeeping gene *Tbp*.

### Western blot

Liver protein isolates were prepared as previously described. (17) 20 ug of protein were loaded for each sample and separated by electrophoresis using NuPage 4–12% Bis-Tris midi gels (Thermo Fisher Scientific, WBT41226BOX) in 1x NuPage MES SDS running buffer (Thermo Fisher Scientific, NP0002) Proteins were transferred to a polyvinylidene fluoride membrane (STM2006) using 1× NuPage Transfer buffer (Thermo Fisher Scientific, NP00061) and blocked in 5% nonfat milk in Tris buffered saline with 0.01% Tween-20 (TBST). Following blocking, antibodies to target proteins (Rabbit anti-PDI (Cell Signaling Technology (CST), #3501, 1:10000), Rabbit anti-ACC (CST, #3676, 1:10000), Rabbit anti-FASN (CST, #3180, 1:10000), and Rabbit anti-SCD1 (CST, #2794, 1:10000) (Supplemental Table S2) were diluted in 5% nonfat milk in TBST and incubated with the membrane overnight at 4° C. Following incubation, membranes were washed with TBST and incubated with donkey anti-rabbit HRP-conjugated secondary antibody at room temperature for one hour. Following washing in TBST, blots were developed with HRP substrate (Thermo Fisher Scientific, WBLUF0500) on Amersham Imager 600 (GE Healthcare, Chicago, IL).

### Bulk RNA-sequencing analysis

Total RNA was extracted from flash-frozen tissue samples using the RNeasy Mini Kit (Qiagen, 74104). For quality control of RNA integrity and library synthesis products, the RNA ScreenTape and D1000 ScreenTape kits were used with the 4200 TapeStation system (Agilent Technologies). mRNA libraries were prepared from 800 ng RNA using the KAPA RNA Hyperprep Kit (KAPA Biosystems, KK8541), with KAPA Unique Duel-Indexed Adaptor Kit (KAPA Biosystems, KK8727). The pooled libraries were then submitted to the UCLA Technology Center for Genomics & Bioinformatics for sequencing. Paired-end sequencing was performed on a NovaSeq X Plus platform (Illumina) in the 2 × 100 bp configuration.

Raw FASTQ files were trimmed for adapter sequences and mapped to the mm10 mouse assembly using STAR (v2.1.1). (31) Gene counts were quantified using featureCounts (Subread v2.1.1). (32) Genes with fewer than 600 total counts across all samples were excluded from downstream analyses. Differential expression analysis was conducted using DESeq2 (33) (v1.38.3) in R (v4.2.1). Genes were determined as differentially expressed between two conditions if they passed these thresholds: −1.0 ≤ log_2_ fold change ≥ 1.0 and adjusted *P*-value ≤ 0.05. Differentially expressed genes were plotted using EnhancedVolcano (v1.16.0), RColorBrewer (v1.1.3), and ggplot2 (v3.5.0) packages in R (v4.2.1). Gene set enrichment analysis (GSEA) was performed using the clusterProfiler (34, 35) package (v4.6.2) with MSigDB gene sets in R (v4.2.1). (36) GSEA hallmark gene set pathways (37) were plotted using ggplot2 (v3.5.0) in R (v4.2.1).

### Statistical analyses

Statistical analyses were performed using Prism (GraphPad v10). All data are represented as mean ± SEM, unless otherwise noted. Physiological measurements were taken from individual biological replicates. A two-sided Student’s *t* test was used for single variable comparison between two groups. A two-way ANOVA followed by Šídák’s or Tukey’s multiple comparisons test was used to examine interactions between multiple variables, as appropriate. A *P*-*value* < 0.05 was considered statistically significant.

## Results

### Intestinal lipase inhibition by orlistat attenuates obesity by promoting fecal fat excretion

**Fig. 1:**
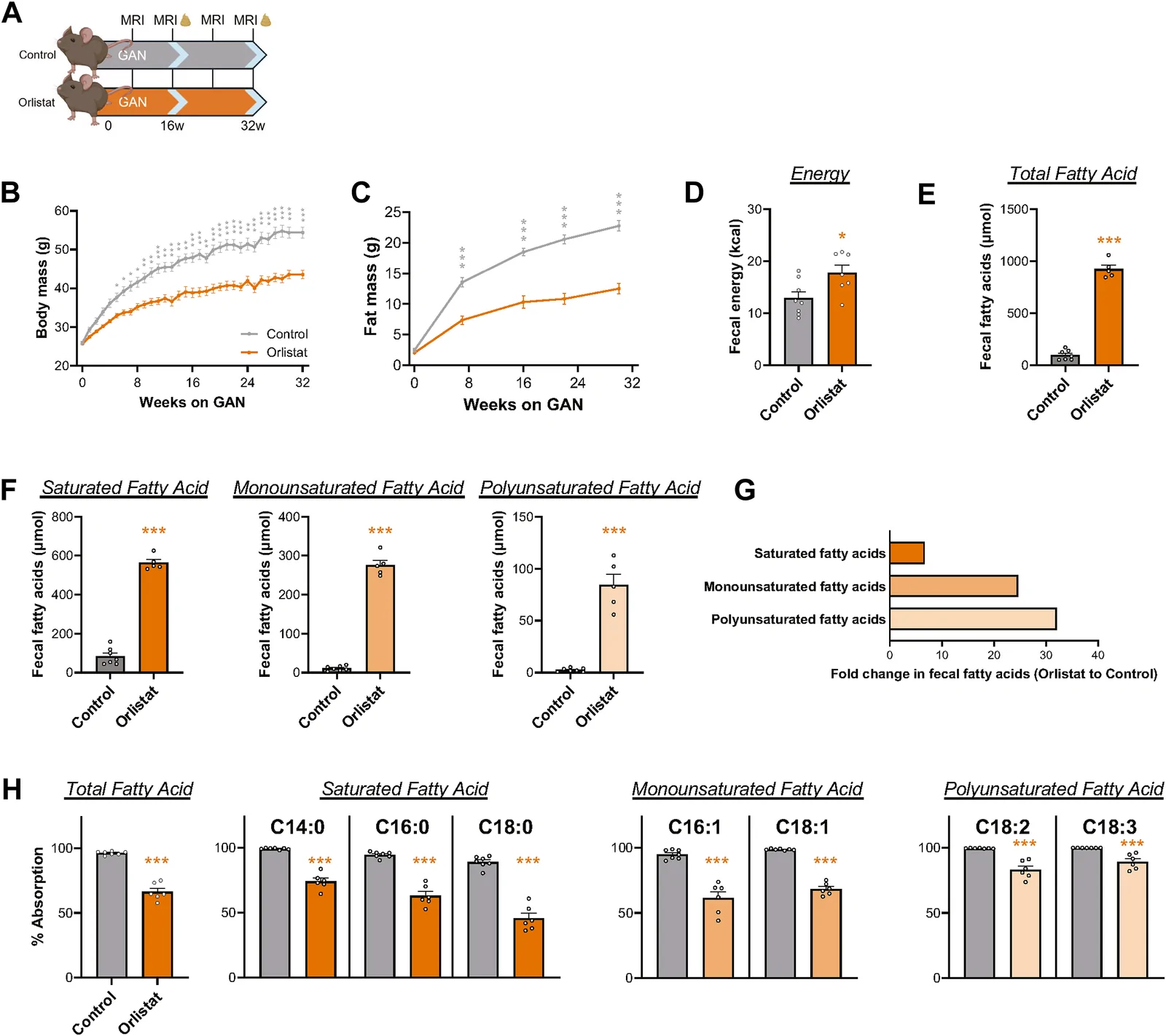
Intestinal lipase inhibition with orlistat attenuates GAN diet-induced obesity by promoting fecal fatty acid excretion. A: Experimental schematic to induce obesity and MASH in control and orlistat-treated mice fed GAN diet for 32 weeks. Feces were collected at weeks 16 and 32 to measure energy and fatty acid excretion. A GAN diet containing a sucrose polybehenate tracer was provided at weeks 16 and 32 (blue chevron) to assess fatty acid absorption. Body composition was assessed by magnetic resonance imaging (MRI) approximately every 8 weeks. B: Body mass for control and orlistat-treated mice over 32 weeks. Data are represented as mean ± standard error of mean (SEM). ∗*P* < 0.05, ∗∗*P* < 0.01, ∗∗∗*P* < 0.001, by two-way ANOVA with Šídák’s multiple comparisons test. *n* = 12 mice per group. C: Fat mass for control and orlistat-treated mice over 32 weeks. Data are represented as mean ± standard error of mean (SEM). ∗∗∗*P* < 0.001, by two-way ANOVA with Šídák’s multiple comparisons test. *n* = 12 mice per group. D: Fecal excretion of total energy over week 32 of experiment. Data are represented as mean ± SEM. ∗*P* < 0.05, by two-sided *t* test. *n* = 7–8 mice per group. E: Fecal excretion of total fatty acids over week 32 of experiment. Data are represented as mean ± SEM. ∗∗∗*P* < 0.001, by two-sided *t* test. Each point represents the average of 5 group-housed mice. *n* = 5–7 mice per group. F: Fecal excretion of saturated, monounsaturated, and polyunsaturated fatty acids over week 32 of experiment. Data are represented as mean ± SEM. ∗∗∗*P* < 0.001, by two-sided *t* test. *n* = 5–7 mice per group. G: Fold change in amounts of fecal saturated, monounsaturated, and polyunsaturated fatty acids in orlistat-treated mice compared to control mice. Fold change values were calculated from average fecal fatty acid excretion within each group. H: Absorption of total, saturated, monounsaturated, and polyunsaturated fatty acids over week 32 of experiment. Data are represented as mean ± SEM. ∗∗∗*P* < 0.001, by two-sided *t* test. *n* = 7 mice per group. GAN, Gubra-Amylin NASH.

The GAN diet is a palm oil-based high-fat diet (Supplemental Fig. S1A) that recapitulates many of the metabolic and histologic hallmarks of human MASH. (38, 39, 40, 41) To determine the role of intestinal lipase inhibition on GAN-induced obesity, mice were fed the GAN diet for 32 weeks with or without orlistat (Fig. 1A). Compared to GAN-fed control mice, orlistat-treated mice exhibited significantly reduced body weight gain (Fig. 1B). This effect was entirely driven by changes in fat mass (Fig. 1C), as lean mass was preserved (Supplemental Fig. S1B).

To investigate the basis for the difference in body mass between groups, we examined fecal energy loss using oxygen bomb calorimetry. Orlistat-treated mice showed a marked increase in total fecal energy excretion after 32 weeks (Fig. 1D), which correlated with a substantial increase in total fecal fatty acid content, measured by GC-MS following methanolysis of fecal lipids (Fig. 1E). Analysis of the fatty acid composition revealed that orlistat-treated mice excreted disproportionately greater amounts of polyunsaturated and monounsaturated fatty acids than saturated fatty acids compared to control mice (Fig. 1F, G). This was surprising, given that unsaturated fatty acids are normally absorbed more efficiently than saturated fatty acids (42, 43).

To directly quantify intestinal absorption, we incorporated SPB as a nonabsorbable dietary lipid tracer into the GAN diet, which facilitates measurements of absorption for individual fatty acids. (28) The absorption of all fatty acids were broadly decreased by orlistat (Figs. 1H and Supplemental Fig. S1C). Although orlistat has been shown to inhibit dietary cholesterol absorption, (44) no differences in fecal cholesterol excretion were found between groups on the GAN diet (Supplemental Fig. S1D), potentially due to the diet’s high cholesterol content. Finally, food intake was not significantly different between groups (Supplemental Fig. S1E), indicating that the observed changes in body mass and fecal excretion were not driven by alterations in energy consumption.

### Lipase inhibition fails to prevent progression of liver disease on ultrasound imaging

Recent advances in automated ultrasound imaging have enabled noninvasive, longitudinal monitoring of liver size, echogenicity, and stiffness in murine models of MASLD. (45, 46) To determine whether orlistat’s protection against obesity also translated to MASH, we used a high-throughput, automated preclinical ultrasound imaging platform (Vega™, Revvity, Inc., Waltham, MA) to longitudinally monitor liver morphology and stiffness during GAN feeding. (45) The platform acquires sequential 2D frames in brightness (B) and SWE modes that are reconstructed into 3D datasets for noninvasive assessment of liver volume, echogenicity, and stiffness in vivo. Liver volumes were quantified using an artificial intelligence-assisted workflow trained on expert-annotated datasets, with all measurements independently confirmed by a human expert.

**Fig. 2:**
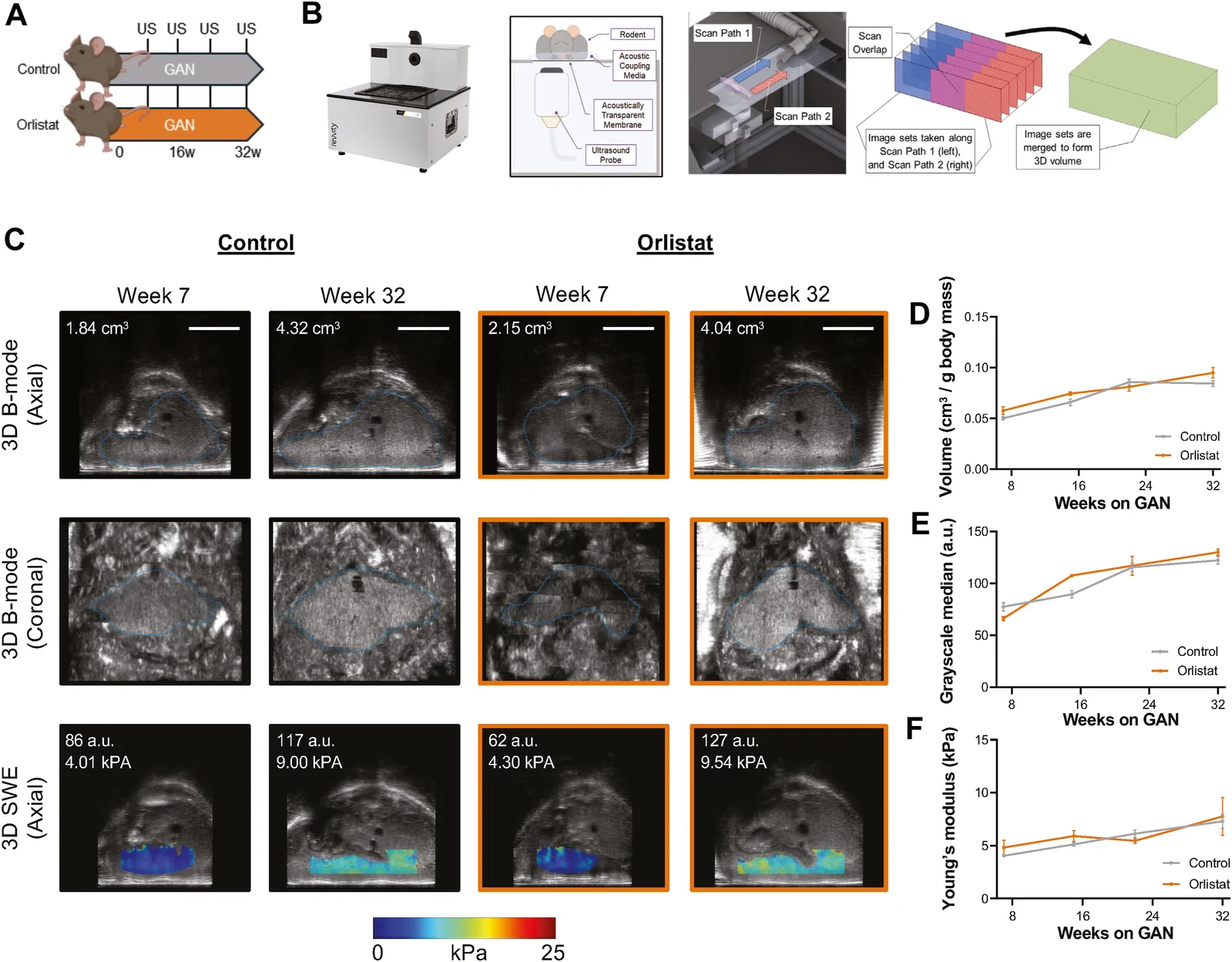
Lipase inhibition fails to prevent progression of liver disease on ultrasound imaging. A: Experimental schematic for noninvasive monitoring of liver disease in GAN-fed control and orlistat-treated mice using automated ultrasound (US) and shear wave elastography (SWE) approximately every 8 weeks over 32 weeks. B: Overview of image acquisition workflow. Mice are placed on the Vega™ ultrasound instrument (Revvity, Inc) in prone position and imaged from below via robotically controlled raster scan, with reconstruction of raw 2D frames into 3D volumes. Livers are scanned in multimodal 3D brightness mode and SWE. C: Representative in vivo images of the liver over the 32 weeks. Axial and coronal slices from 3D brightness (B)-mode images depicting liver size and echogenicity are shown in the top and middle rows, while axial plane from 3D SWE images depicting liver stiffness in Young's modulus are shown in the bottom row. The scale bar represents 1 cm. (D) Liver volume, (E) echogenicity, and (F) stiffness in control and orlistat-treated mice fed GAN diet at interval timepoints over 32 weeks *n* = 2–4 mice per time point per group. GAN, Gubra-Amylin NASH.

To track MASH progression, control and orlistat-treated mice were imaged longitudinally at approximately 8-week intervals over 32 weeks using automated ultrasound and SWE (Fig. 2A, B). Liver volume, liver echogenicity, and liver stiffness served as noninvasive surrogates of hepatomegaly, steatosis, and fibrosis, respectively.

Serial longitudinal imaging revealed progressive increases in liver volume and echogenicity during GAN feeding, consistent with the development of hepatomegaly and steatosis in all mice (Fig. 2C–E, Supplemental Fig. S2A, B). Liver stiffness also increased gradually across the study period, consistent with mild to moderate fibrosis (Fig. 2F). Despite marked reductions in body fat mass with orlistat treatment (Fig. 1C), it was particularly surprising that no differences were detected between groups at any time point. Collectively, these longitudinal in vivo imaging data demonstrate that pharmacologic inhibition of dietary fatty acid absorption does not prevent GAN diet-induced increases in liver size, steatosis, or fibrosis, highlighting a dissociation between obesity and liver disease progression with orlistat.

### Hepatic inflammation persists with intestinal lipase inhibition despite reduced weight gain

**Fig. 3:**
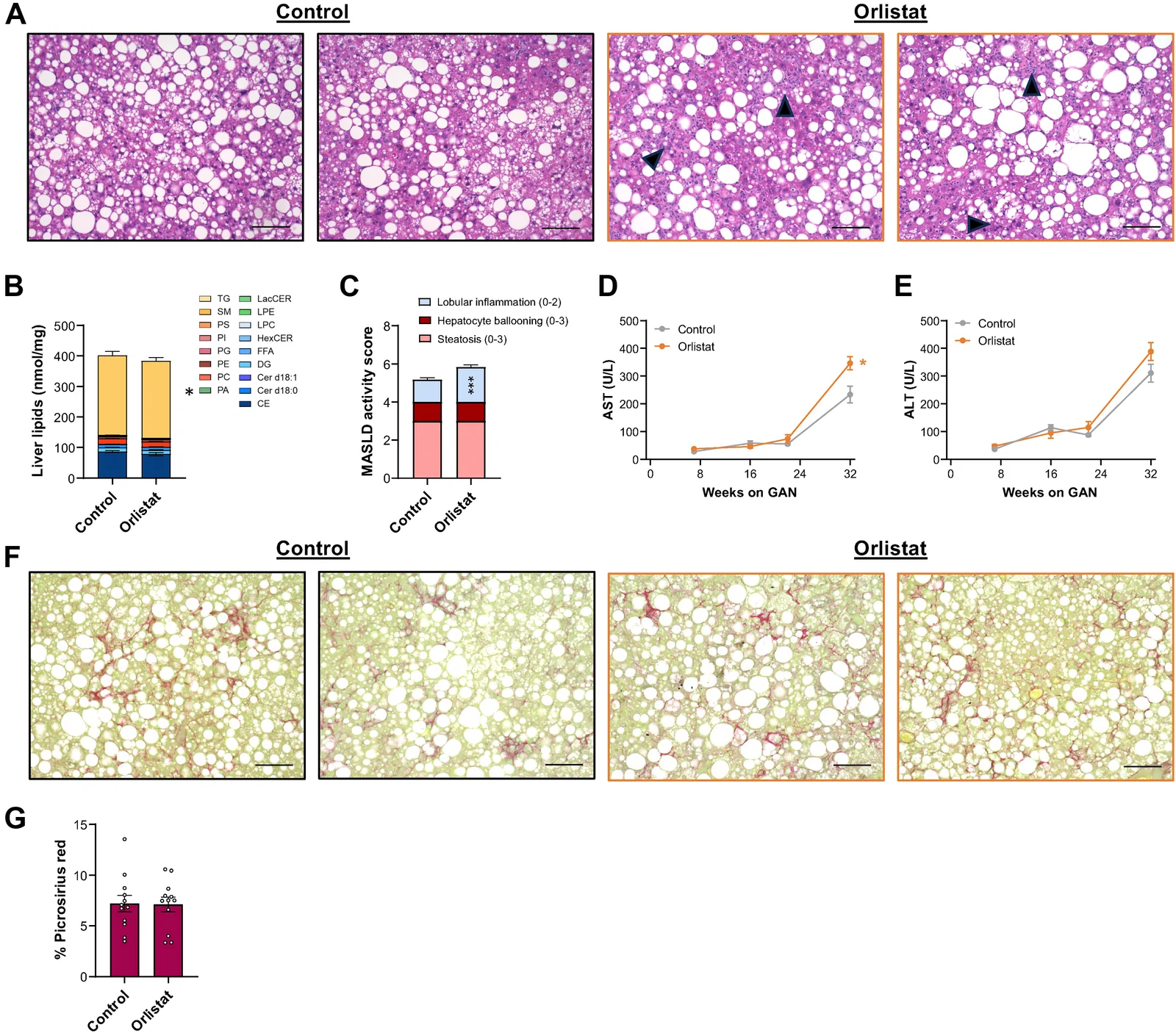
Hepatic inflammation persists with lipase inhibition despite reduced weight gain. A: Histological images of representative liver sections stained with hematoxylin and eosin from control (black box) and orlistat-treated (orange box) mice after 32 weeks. Regions of increased inflammatory cell infiltration are marked by black triangle. The scale bar represents 100 μm. B: Lipidomic analyses of liver lipid classes from control and orlistat-treated mice after 32 weeks. *n* = 8 mice per group. Data are represented as mean ± SEM. ∗*P* < 0.05, by multiple *t* tests between lipid classes. C: Histological MASLD activity score in control and orlistat-treated mice after 32 weeks. Data are represented as mean ± SEM. ∗∗∗*P* < 0.001, by multiple t-tests. *n* = 12 mice per group. (D) Plasma AST and (E) plasma ALT levels in control and orlistat-treated mice fed GAN diet over 32 weeks. Data are represented as mean ± SEM. ∗*P* < 0.05, by two-way ANOVA with Šídák’s multiple comparisons test. *n* = 10–12 mice per group. F: Histological images of representative liver sections stained with Picrosirius red from control (black box) and orlistat-treated (orange box) mice after 32 weeks. The scale bar represents 100 μm. G: Quantification of Picrosirius red-positive area in liver sections from control and orlistat-treated mice after 32 weeks. Data are shown as percentage of total liver area. Data are represented as mean ± SEM. n = 12 mice per group. TG, triglyceride; CE, cholesterol ester; Cer d18:0, ceramide 18:0; Cer d18:1, ceramide 18:1; DG, diacylglyceride; FFA, free fatty acid; HexCER, hexosylceramide; LacCER, lactosylceramide; LPC, lysophosphatidylcholine; LPE, lysophosphatidylethanolamine; MASLD, metabolic dysfunction-associated steatotic liver disease; PA, phosphatidic acid; PC, phosphatidylcholine; PE, phosphatidylethanolamine; PG, phosphatidylglycerol; PI, phosphatidylinositol; PS, phosphatidylserine; SM, sphingomyelin.

We next examined whether the in vivo imaging findings correlated with endpoint liver histopathology. After 32 weeks of GAN feeding, orlistat-treated mice showed no difference in liver weight compared with controls (Supplemental Fig. S3A). Histological analysis of H&E-stained liver sections showed that both groups exhibited marked macrovesicular steatosis and hepatocyte ballooning, a key feature of steatohepatitis (Fig. 3A). Steatosis was confirmed by hepatic lipidomic analysis, with no differences across major hepatic lipid classes between groups, except for phosphatidic acid (Fig. 3B). Notably, hepatic triglyceride levels—the dominant lipid species in MASLD livers—were unchanged despite reduced intestinal fatty acid absorption. Consistent with the lack of change in dietary cholesterol absorption, hepatic cholesterol levels also did not differ between groups, likely reflecting the supraphysiologic cholesterol load in the GAN diet. Histologically, lobular inflammation was modestly increased in orlistat-treated livers, with denser inflammatory cell infiltration (Fig. 3A). The MASLD activity score (0–8), a composite measure based on steatosis (0–3), lobular inflammation (0–3), and hepatocyte ballooning (0–2), was higher in orlistat-treated mice (Fig. 3C), suggesting that lipase inhibition did not mitigate disease activity. This was accompanied by biochemical evidence of hepatocellular injury, with elevated plasma aminotransferases AST and ALT. While they were only modestly increased at earlier time points, they increased abruptly in the final weeks (Fig, 3D, E), indicating that liver injury emerged predominantly at late stages after a prolonged period of cumulative stress. This trajectory is consistent with prior reports that extended GAN diet exposure is required to induce steatohepatitis. (39) However, it was unexpected that orlistat exacerbated liver inflammation, given the protection from weight gain.

**Fig. 4:**
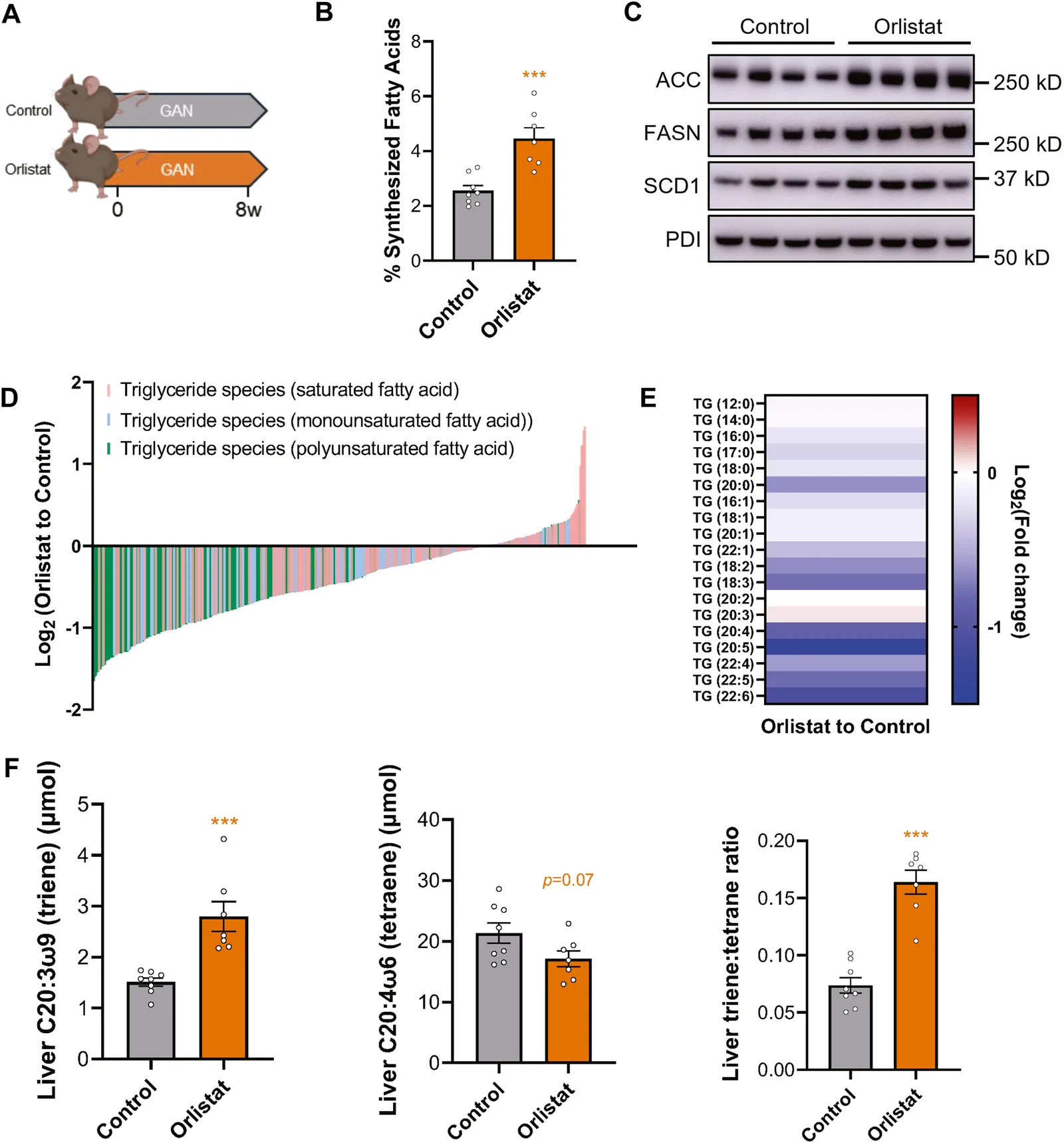
Lipase inhibition depletes hepatic polyunsaturated fatty acids and activates de novo lipogenesis. A: Experimental schematic to induce obesity and hepatic steatosis in control and orlistat-treated mice fed GAN diet for 8 weeks. B: Percentage of total newly synthesized deuterium-labeled fatty acids in control and orlistat-treated mice after 8 weeks. Data are represented as mean ± SEM. ∗∗∗*P* < 0.001, by two-sided *t* test. *n* = 7–8 mice per group. C: Western blot analyses of hepatic acetyl-CoA carboxylase (ACC), fatty acid synthase (FASN), and stearoyl-CoA desaturase 1 (SCD1) expression in representative control and orlistat-treated mice after 8 weeks. D: Lipidomic analysis of the abundance of liver triglyceride species containing saturated fatty acids (SFA), monounsaturated fatty acids (MUFA), and polyunsaturated fatty acids (PUFA), shown as log fold change in orlistat-treated mice relative to controls. *n* = 8 mice per group. E: Heatmap of the mean concentration of liver triglyceride species containing a specific fatty acid, shown as fold change in orlistat-treated mice relative to controls. *n* = 8 mice per group. F: Liver mead acid (C20:3ω9), arachidonic acid (C20:4ω6), and triene:tetraene ratio in control and orlistat-treated mice after 8 weeks. Data are represented as mean ± SEM. ∗∗∗*P* < 0.001, by two-sided *t* test. *n* = 7–8 mice per group. GAN, Gubra-Amylin NASH.

**Fig. 5:**
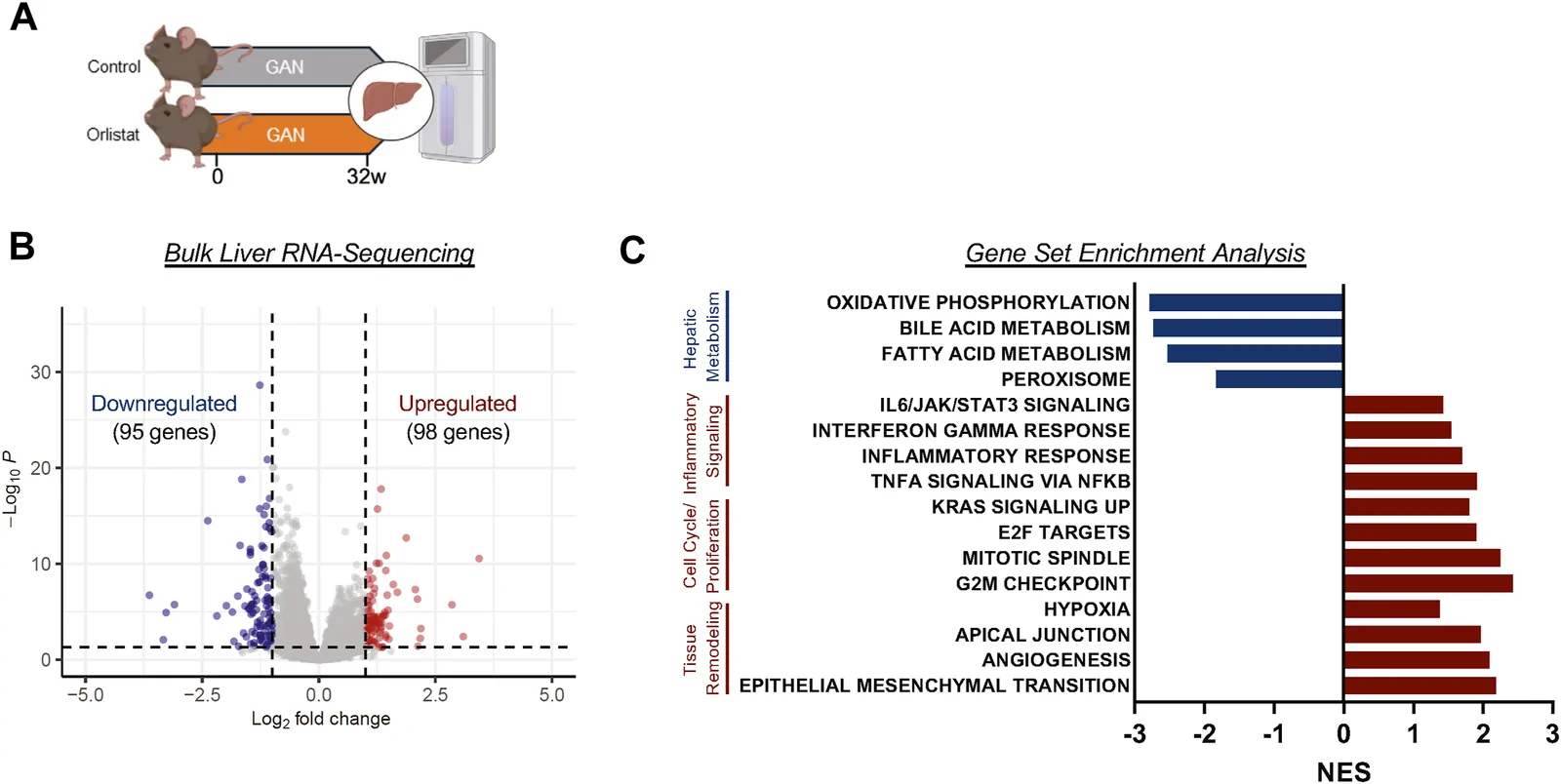
Lipase inhibition is associated with maladaptive hepatic transcriptional reprogramming. A: Experimental schematic of bulk RNA sequencing performed on liver tissues from orlistat-treated and control mice after 32 weeks. B: Volcano plot of differentially expressed genes in bulk liver RNA-sequencing from orlistat-treated and control mice after 32 weeks *n* = 6 mice per group. Horizontal dashed line is the threshold at which differences reach statistical significance. Vertical dashed lines represent log2 fold change thresholds at < −1 for downregulated genes and > 1 for upregulated genes. Genes that are significantly downregulated are colored blue, genes that are significantly upregulated are colored red, genes that are not significant are colored gray. Statistical significance was determined using DESeq2 (*p*-adjusted < 0.05). C: Gene set enrichment analysis (GSEA) of bulk liver RNA-sequencing data from orlistat-treated mice compared to controls after 32 weeks. *n* = 6 mice per group. Significantly enriched pathways (*p*-adjusted < 0.05) with normalized enrichment score (NES) > 1.5 or < 1.5 were selected for visualization and grouped into biological themes.

Liver fibrosis, assessed by picrosirius red staining of collagen deposition, was mild-to-moderate and comparable between groups (Fig. 3F, G), in line with the extent of fibrosis from other reports using the GAN diet. (38, 39) Together, these findings demonstrate that, despite reducing body weight, orlistat failed to protect against hepatic steatosis and fibrosis and increased inflammatory liver injury.

### Lipase inhibition depletes hepatic PUFAs and activates de novo lipogenesis

Given the marked decrease in intestinal fatty acid absorption, we wondered why hepatic triglyceride accumulation persisted. Because de novo lipogenesis is a major contributor to hepatic triglyceride pools, (47) we assessed lipogenic activity in the liver. We first measured mRNA of key lipogenic enzymes, including acetyl-CoA carboxylase (*Acc1*), fatty acid synthase (*Fasn*), and stearoyl-CoA desaturase-1 (*Scd1*) (Supplemental Fig. S3B), and directly quantified de novo lipogenesis in vivo using deuterium labeling of newly synthesized fatty acids (Supplemental Fig. S3C), as described previously. (25) At week 32, lipogenesis trended higher in orlistat-treated mice but did not reach statistical significance. At the same time, we measured hepatic cholesterol synthesis and found no differences between groups (Supplemental Fig. S3D). We reasoned that the chronic high-fat intake may have dampened lipogenic pathways at this advanced disease stage, possibly masking increases in de novo lipogenesis that initiated hepatic triglyceride accumulation.

Thus, we repeated the studies using the GAN diet with and without orlistat, but collected tissues at an earlier time point to capture lipid changes that might have preceded the development of severe steatosis (Fig. 4A). At 8 weeks, orlistat-treated mice were already protected against diet-induced obesity (Supplemental Fig. S4A), with no differences in liver size (Supplemental Fig. S4B). Total hepatic triglyceride levels were also similar between groups (Supplemental Fig. S4C). However, direct measurement of de novo lipogenesis revealed significantly increased synthesis of hepatic fatty acids in orlistat-treated mice (Figs. 4B, Supplemental Fig. S4D). Together with the increased mRNA (Supplemental Fig. S4E) and protein expression of ACC, FASN, and SCD1 (Fig. 4C), these findings indicate that hepatic lipogenesis was activated early and preceded overt triglyceride accumulation at later stages. Meanwhile, total hepatic cholesterol levels (Supplemental Fig. S4F) and newly synthesized cholesterol (Supplemental Fig. S4G) did not differ between groups, even at this early time point.

Because PUFAs are known to regulate hepatic lipogenesis, (13, 48, 49) we next examined whether alterations in hepatic lipid composition contributed to the increased lipogenic activity observed in orlistat-treated mice. Under normal conditions, PUFAs suppress lipogenesis by inhibiting the proteolytic activation of sterol regulatory element-binding protein-1c (SREBP-1c), the master regulator of lipid homeostasis. (50) When hepatic PUFAs are deficient, this suppressive effect is lost, leading to increased SREBP-1c activity and de novo lipogenesis. Lipidomic analysis revealed a reduction in PUFA-containing triglyceride species and a reciprocal increase in triglycerides enriched in saturated fatty acids in orlistat-treated mice (Fig. 4D). Because insulin resistance can also activate hepatic lipogenesis, (50) we assessed glucose tolerance but found no sign of insulin resistance (Supplemental Fig. S4H), suggesting the changes in lipogenesis are driven by differences in PUFA.

Among triglycerides containing specific fatty acid species that were altered in orlistat treated mice, C20:3-containing triglycerides were increased, whereas all others were decreased (Fig. 4E). We confirmed the increase in total liver C20:3 by GC-MS analysis (Fig. 4F). This increase in C20:3 was of particular significance, because it was attributable to Mead acid (C20:3ω-9) and accompanied by a decrease in arachidonic acid (C20:4ω-6) (Fig. 4F). Normally, the essential fatty acid linoleic acid (C18:2ω-6) is desaturated and elongated to generate arachidonic acid. However, when essential PUFAs are depleted, the same enzymes are redirected toward oleic acid (C18:1ω-9), resulting in the synthesis and accumulation of Mead acid (C20:3ω-9) (Supplemental Fig. S4I). (51) Clinically, an essential fatty acid deficiency is defined by the ratio of Mead acid (triene) to arachidonic acid (tetraene) exceeding 0.2. (52) Although the triene:tetraene ratio did not reach this threshold, it was significantly higher in orlistat-treated mice compared to control mice, consistent with a relative PUFA deficiency (Figs. 4F, Supplemental Fig. S4J). Thus, the enhanced hepatic lipogenesis observed in orlistat-treated mice was most likely due to the decrease in key PUFAs. Taken together, these data indicate that the malabsorption of PUFAs by orlistat leads to an essential fatty acid deficiency, promoting hepatic lipogenesis and limiting protection against steatosis.

### Lipase inhibition is associated with maladaptive hepatic transcriptional reprogramming

PUFA deficiency has been shown to impair the generation of PUFA-derived lipid mediators that actively promote resolution of inflammation. (12, 53) As obligate precursors for specialized pro-resolving mediators, reduced PUFA availability can alter transcriptional programs involved in metabolic homeostasis. (12) To determine whether hepatic PUFA depletion (Supplemental Fig. S5A, B) was accompanied by broader transcriptional changes within the liver, we next performed bulk RNA sequencing on liver tissue from control and orlistat-treated mice after 32 weeks of GAN diet feeding (Fig. 5A). Transcriptomic profiling identified 95 significantly downregulated and 98 upregulated genes between groups (Fig. 5B). GSEA (37) revealed transcriptional programs related to hepatic metabolism, cell-cycle and proliferation, inflammatory signaling, and tissue remodeling (Fig. 5C, Supplemental Table S3).

Among the negatively enriched pathways, GSEA demonstrated marked suppression of oxidative phosphorylation, fatty acid metabolism, bile acid metabolism, and peroxisome pathways, indicating downregulation of hepatic metabolism in orlistat-treated mice, likely a consequence of metabolic dysfunction associated with orlistat use. At the same time, cell-cycle and proliferative programs, including G2M checkpoint, mitotic spindle, E2F targets, and KRAS signaling pathways, were significantly enriched. Orlistat also upregulated inflammatory signaling pathways, including TNFα signaling via NFκB, inflammatory response, interferon-γ response, and IL6/JAK/STAT3 signaling. These pathways promote immune cell recruitment, cytokine production, and sustained hepatic inflammation and are well-established drivers in the transition from simple steatosis to steatohepatitis. (54) Activation of these pathways may reflect a compensatory regenerative response to chronic metabolic stress and hepatocellular injury (Fig. 3C–E). Finally, pathways associated with extracellular matrix remodeling and tissue injury responses, including epithelial-mesenchymal transition, angiogenesis, apical junction, and hypoxia were significantly enriched in orlistat-treated mice. These transcriptional programs are commonly associated with fibrogenic remodeling and disease progression in MASH. (55) To validate the transcriptional enrichment of fibrogenic pathways, we examined hepatic expression of select profibrotic genes by quantitative PCR. At 32 weeks, orlistat-treated mice exhibited increased hepatic expression of *Timp1*, *Col1a1*, and *Tgfb1* (Supplemental Fig. S5C), indicating activation of pro-fibrogenic pathways. Although differences in histologic fibrosis were not yet apparent (Fig. 3G, H), these findings suggest that early transcriptional changes may predispose to worse fibrosis over longer disease duration. Collectively, these findings suggest that orlistat-induced alterations in hepatic lipid composition are accompanied by coordinated transcriptional reprogramming across metabolic, inflammatory, and fibrogenic pathways relevant to MASH progression.

## Discussion

In this study, we show that intestinal lipase inhibition with orlistat protects against obesity but fails to ameliorate, and instead exacerbates, MASH in a GAN diet-induced MASH mouse model. Consistent with its mechanism of action, orlistat reduced net energy absorption by decreasing dietary fatty acid absorption. However, this benefit came at the expense of nonselective inhibition of fatty acid absorption, broadly reducing uptake of all fatty acid species, including PUFAs. Because hepatic lipids are derived largely from dietary fatty acids, (47) the disproportionate excretion of PUFAs relative to saturated fatty acids depleted the liver of key PUFAs, reshaping the hepatic lipidome and effectively uncoupling improvements in obesity from protection against MASH.

To our knowledge, this is one of few studies to integrate automated 3D ultrasound imaging with SWE for longitudinal assessment in a dietary MASH model. (46, 56, 57) Using the Vega ultrasound system, we performed noninvasive, real-time monitoring of liver volume, echogenicity, and stiffness over time, with findings corroborated by endpoint histology. This platform allowed detection of divergence between weight trajectory and liver pathology throughout disease progression, further validating the potential role of ultrasound imaging for monitoring in preclinical models.

The modest protection against obesity observed with orlistat in this study aligns with human data. In clinical trials, orlistat produces small but reproducible reductions in body weight when combined with either a low-fat diet or physical activity. (20, 21) However, both preclinical and clinical studies evaluating its effects on MASLD have yielded inconsistent results, likely due to the heterogeneity in study design and clinical context. (58, 59, 60, 61, 62) Several animal studies using different high-fat diet models have reported improvements in hepatic steatosis with orlistat treatment. (62, 63) However, in a seminal randomized controlled trial, orlistat was not shown to improve liver histology beyond diet alone, with histologic improvement correlating more closely with the magnitude of weight loss than with orlistat treatment itself. (59) Meta-analyses similarly report mild reductions in liver enzymes but fail to consistently improve liver histology (64).

Our data show that liver inflammation persisted in the orlistat-treated mice despite modest reductions in body weight, highlighting a dissociation between weight loss and liver health. The discrepancy between our findings and those of prior studies may reflect differences in the dietary context in which intestinal lipase inhibition was implemented. The GAN diet used in our experiments contains 20% fructose, which was absent from the diets used in prior studies. While fructose is a well-established driver of MASLD, (65) orlistat does not inhibit fructose absorption. (19) Thus, the fructose component of the diet is unlikely to account for the worse hepatic injury observed in orlistat-treated mice. The GAN diet, however, contains a moderate amount of PUFAs. Our fecal fatty acid analysis revealed that orlistat disproportionately promotes the loss of PUFAs relative to saturated fatty acids, leading to depletion of hepatic PUFA stores. Consistent with our findings, a previous study in humans also demonstrated that orlistat reduced the proportion of linoleic acid in serum cholesterol esters, triglycerides, and phospholipids. (66) This shift in fatty acid composition may have significant implications in hepatic lipid metabolism and MASLD progression. Lipidomic analysis of livers from MASLD patients by Puri and colleagues reported progressive reductions in long-chain PUFAs, including arachidonic acid (20:4ω-6), eicosapentaenoic acid (20:5ω-3) and docosahexaenoic acid (22:6ω-3), from healthy controls to patients with MASLD and MASH. (8) Mechanistically, depletion of hepatic PUFAs promotes de novo lipogenesis through loss of PUFA-mediated suppression of SREBP-1c. (13, 48, 49) Altogether, these findings suggest that the efficacy of orlistat may depend on the dietary fatty acid composition in which it is used, underscoring the importance of dietary context in its clinical application. Future studies examining the impact of orlistat on postprandial lipid composition will be important to further delineate how intestinal lipase inhibition directly remodels circulating lipid pools.

In recent work, we showed that reducing the bile acid pool in mice through loss of CYP7A1, the rate-limiting enzyme in hepatic bile acid synthesis, selectively reduces intestinal absorption of saturated fatty acids while largely preserving PUFA uptake. (17) This led to protection against both diet-induced obesity and hepatic steatosis. Because bile acids micellize PUFAs more efficiently than saturated fatty acids, reductions in bile acids disproportionately impair saturated fatty acid absorption. Supporting this, studies of apical sodium-dependent bile acid transporter (*Slc10a2*) inhibition, which reduces the bile acid pool size by blocking enterohepatic bile acid reabsorption, similarly demonstrate that metabolic benefits are driven by dietary fatty acid composition. (67) In these models, metabolic benefits are strongest on saturated fatty acid-rich diets and attenuated on PUFA-enriched diets due to preferential impairment of saturated fatty acid absorption. Consistent with these observations, we previously found that orlistat failed to prevent obesity in mice fed a Western diet, whereas in the present study, the same intervention limited weight gain on a GAN diet despite comparable fat content, suggesting the role of dietary composition beyond total fat. (18) Thus, unlike bile acid-based strategies that selectively alter fatty acid absorption, intestinal lipase inhibition may have a more limited therapeutic window depending on the diet. Future studies should examine lipase inhibition across diets varying in saturated and PUFA content to determine how dietary composition modifies the therapeutic response in MASH.

Taken together, these data identify intestinal fatty acid absorption as a key determinant of hepatic lipid composition and MASH progression. While reducing caloric intake or total fat absorption may modestly improve body weight, indiscriminate blockade of fatty acid uptake can carry unintended hepatic consequences. These findings show that therapeutic outcomes in MASLD depend on both the quantity and composition of absorbed fat and underscore the critical role of selective PUFA uptake in liver health.

## Data availability

The data supporting this study are available in the article, the supplemental data, or from the corresponding author upon request. All original code is available here: https://github.com/tarlingvallimlab/Orlistat-MASH. Raw sequencing data are available on NCBI GEO via the accession code GSE333221 (https://www.ncbi.nlm.nih.gov/geo/query/acc.cgi?acc=GSE333221) for bulk liver RNA-sequencing.

## Supplemental data

This article contains supplemental data.

## Conflict of interest

T. J. C., Z. M., and J. K. P. are employed by Revvity, Inc. All other authors declare that they have no conflicts of interest with the contents of this article.

## Supplemental data

Supplementary Material Supplemental Fig. S1: Intestinal lipase inhibition with orlistat attenuates GAN diet–induced obesity by promoting fecal fatty acid excretion. (A) Fatty acid composition of GAN diet. (B) Lean mass for control and orlistat-treated mice over 32 weeks. Data are represented as mean ± SEM. n = 12 mice per group. (C) Absorption of total, saturated, monounsaturated, and polyunsaturated fatty acids over week 16 of experiment. Data are represented as mean ± SEM. ∗∗∗*p* < 0.001, by two-sided *t* test. n = 6–7 mice per group. (D) Fecal excretion of cholesterol over week 32 of experiment. Data are represented as mean ± SEM. n = 7 mice per group. (E) Average daily food intake per mouse over weeks 16 and 32 of experiment. Each point represents the average of 5 group-housed mice. n = 10 mice per group. GAN, Gubra-Amylin NASH. Supplemental Fig. S2: Lipase inhibition fails to prevent progression of liver disease on ultrasound imaging. (A) Body mass for control and orlistat-treated mice in the longitudinal ultrasound cohort over 32 weeks. Data are represented as mean ± SEM. ∗*p* < 0.05, ∗∗∗*p* < 0.001, by two-way ANOVA with Šídák’s multiple comparisons test. n = 2–4 mice per group. (B) Absolute liver volume in control and orlistat-treated mice at interval time points over 32 weeks ∗*p* < 0.05, ∗∗*p* < 0.01, by two-way ANOVA with Šídák’s multiple comparisons test. n = 2–4 mice per group. Supplemental Fig. S3: Hepatic inflammation persists with lipase inhibition despite reduced weight gain. (A) Absolute liver mass and relative liver mass in control and orlistat-treated mice after 32 weeks. Data are represented as mean ± SEM. n = 12 mice per group. (B) Liver mRNA expression of *Acc1*, *Fasn*, and *Scd1* in control and orlistat-treated mice after 32 weeks n = 12 mice per group. (C) Percentage of total newly synthesized deuterium-labeled fatty acids in control and orlistat-treated mice after 32 weeks. Data are represented as mean ± SEM. n = 12 mice per group. (D) Percentage of newly synthesized deuterium-labeled cholesterol in control and orlistat-treated mice after 32 weeks. Data are represented as mean ± SEM. n = 8–9 mice per group. Supplemental Fig. S4: Lipase inhibition depletes hepatic polyunsaturated fatty acids and activates de novo lipogenesis. (A) Body mass for control and orlistat-treated mice over 8 weeks. Data are represented as mean ± SEM. ∗*p* < 0.05, by two-way ANOVA with Šídák’s multiple comparisons test. n = 16–20 mice per group. (B) Absolute liver mass and relative liver mass in control and orlistat-treated mice after 8 weeks. Data are represented as mean ± SEM. n = 16–18 mice per group. (C) Lipidomic analysis of total liver triglyceride levels in control and orlistat-treated mice after 8 weeks. Data are represented as mean ± SEM. n = 8 mice per group. (D) Percentage of individual newly synthesized deuterium-labeled fatty acids in control and orlistat-treated mice after 8 weeks. Data are represented as mean ± SEM. ∗∗*p* < 0.01, ∗∗∗*p* < 0.001, by two-sided *t* test. n = 7–8 mice per group. (E) Liver mRNA expression of *Acc1*, *Fasn*, and *Scd1* in control and orlistat-treated mice after 8 weeks ∗∗*p* < 0.01, by two-sided *t* test. n = 9–10 mice per group. (F) Hepatic cholesterol level in control and orlistat-treated mice after 8 weeks. Data are represented as mean ± SEM. n = 5–7 mice per group. (G) Percentage of newly synthesized deuterium-labeled cholesterol in control and orlistat-treated mice after 8 weeks. Data are represented as mean ± SEM. n = 5–7 mice per group. (H) Oral glucose tolerance test in control and orlistat-treated mice after 8 weeks. Area under the curve (AUC) is depicted as a bar chart (*right*). n *=* 8 mice per group. (I) Diagram illustrating the metabolism of ω-3, ω-6, and ω-9 polyunsaturated fatty acids. (J) Plasma triene:tetraene ratio in control and orlistat-treated mice after 8 weeks. Data are represented as mean ± SEM. ∗∗∗*p* < 0.001, by two-sided *t* test. n = 8 mice per group. Supplemental Fig. S5: Lipase inhibition is associated with maladaptive hepatic transcriptional reprogramming. (A) Liver C18:2 and C18:3 concentrations in control and orlistat-treated mice after 32 weeks. Data are represented as mean ± SEM. ∗*p* < 0.05, ∗∗∗*p* < 0.001, by two-sided *t* test. n = 8 mice per group. (B) Liver triene:tetraene ratio in control and orlistat-treated mice after 32 weeks. Data are represented as mean ± SEM. ∗∗*p* < 0.01, by two-sided *t* test. n = 7 mice per group. (C) Liver mRNA expression of fibrogenic genes in control and orlistat-treated mice after 32 weeks. Data are represented as mean ± SEM. ∗*p* < 0.05, ∗∗*p* < 0.01, and ∗∗∗*p* < 0.001, by two-sided *t* test. n = 12 mice per group.

Supplemental Tables

Supplemental Table3
