# Supplementary material for: Intestinal lipase inhibition uncouples diet-induced obesity from MASH by remodeling the hepatic lipidome in mice
Source: J Lipid Res. 2026 Jul 27;67(8):101112. doi: 10.1016/j.jlr.2026.101112 (PMC13521235; doi:10.1016/j.jlr.2026.101112)
Supplement: Supplementary file 1 [file mmc1.pdf]

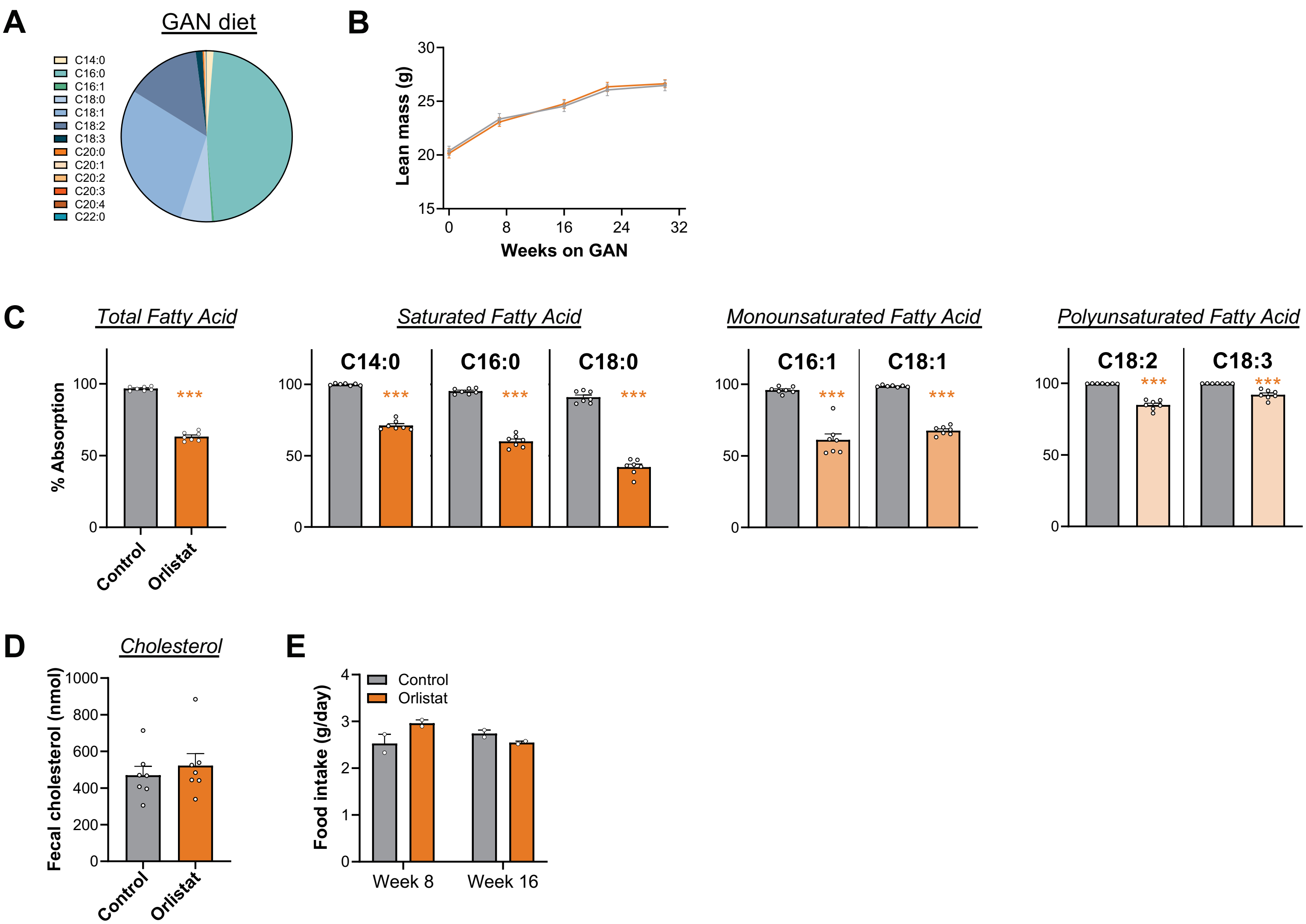

Supplemental Figure 1: Intestinal lipase inhibition with orlistat attenuates GAN diet-induced obesity by promoting fecal fatty acid excretion.

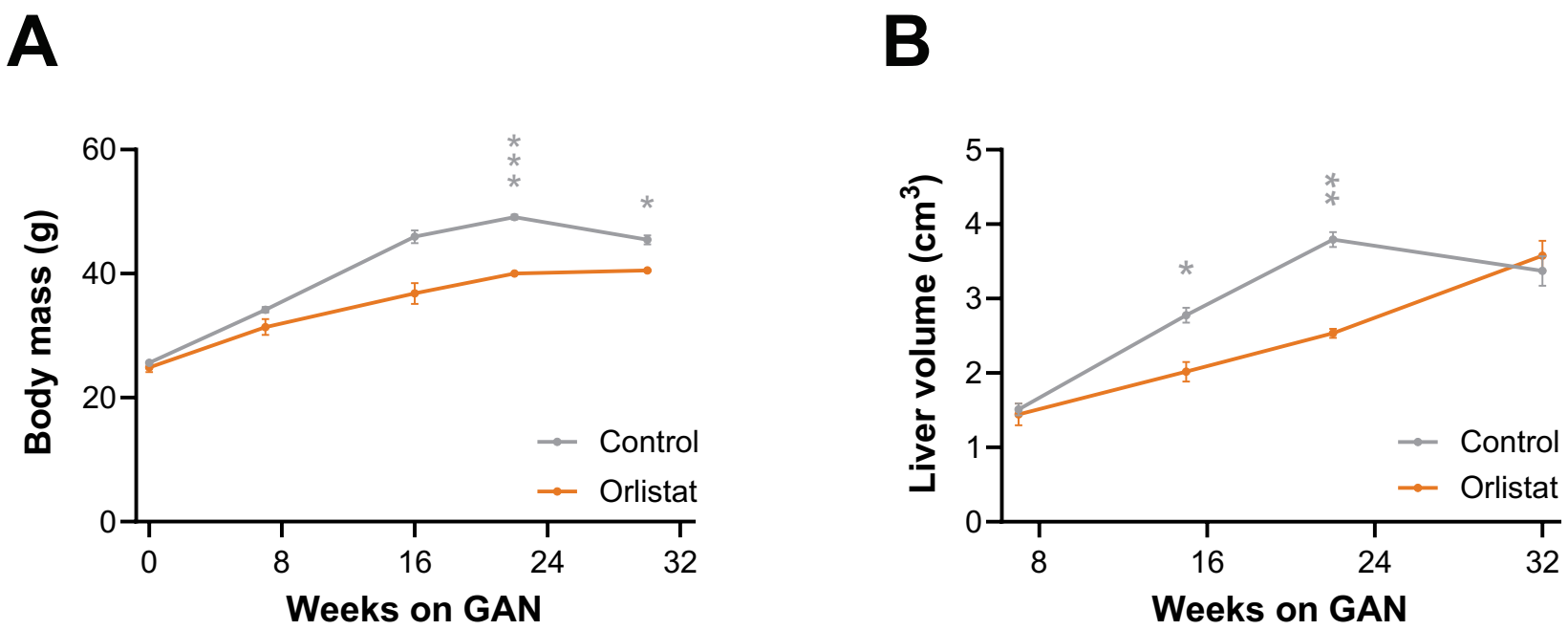

Supplemental Figure 2: Lipase inhibition fails to prevent progression of liver disease on ultrasound imaging.

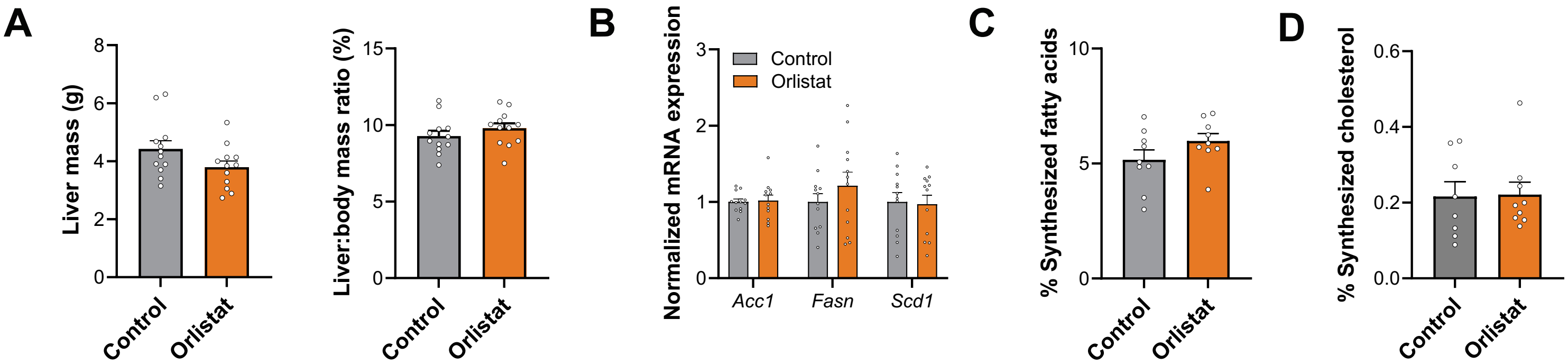

Supplemental Figure 3: Hepatic inflammation persists with lipase inhibition despite reduced weight gain.

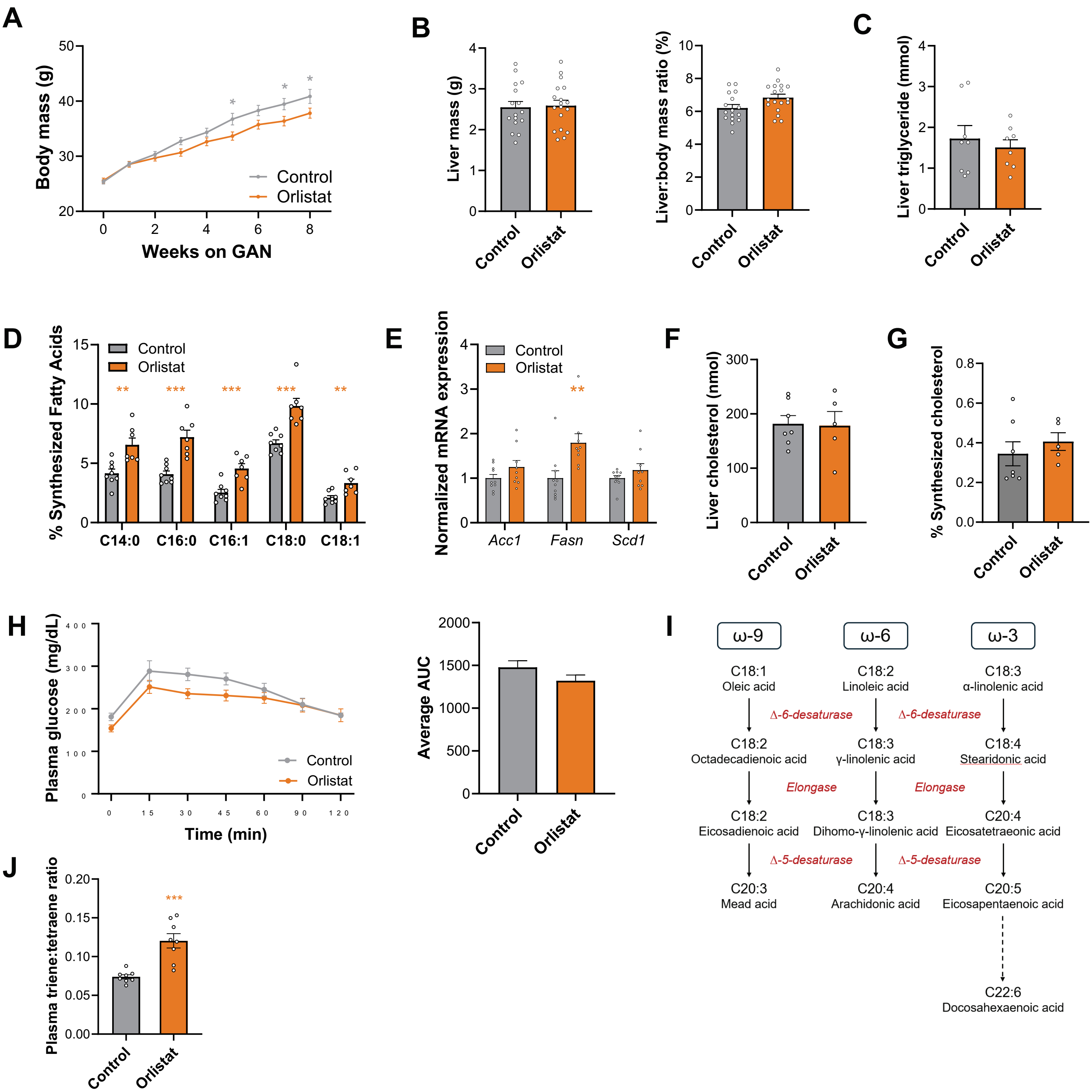

Supplemental Figure 4: Lipase inhibition depletes hepatic polyunsaturated fatty acids and activates *de novo* lipogenesis.

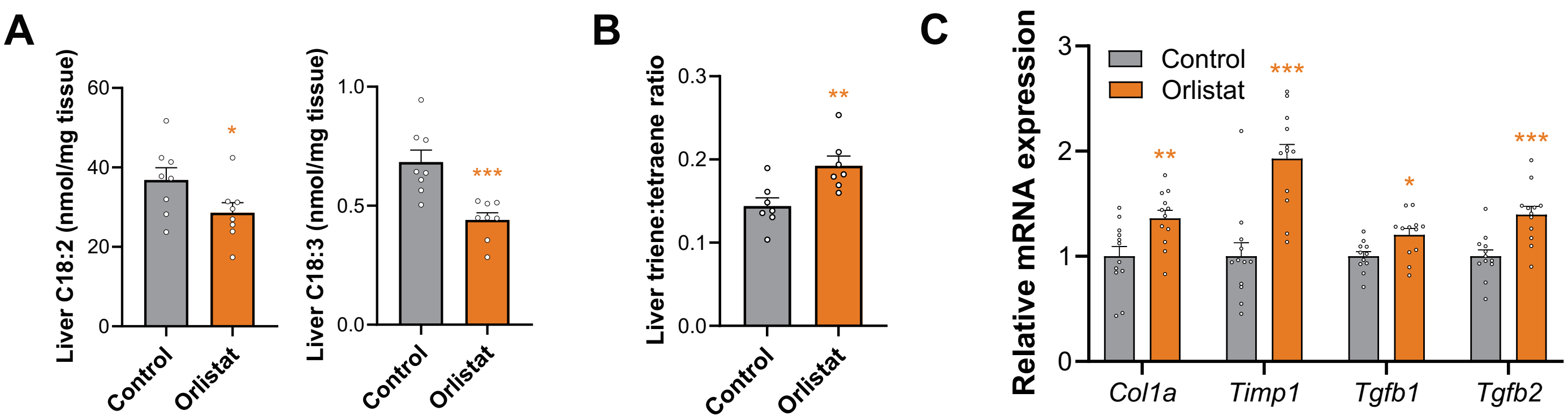

Supplemental Figure 5: Lipase inhibition is associated with maladaptive hepatic transcriptomic reprogramming.
