# Supplementary material for: Intestinal lipase inhibition uncouples diet-induced obesity from MASH by remodeling the hepatic lipidome in mice
Source: J Lipid Res. 2026 Jul 27;67(8):101112. doi: 10.1016/j.jlr.2026.101112 (PMC13521235; doi:10.1016/j.jlr.2026.101112)
Supplement: Supplementary file 2 [file mmc2.pdf]

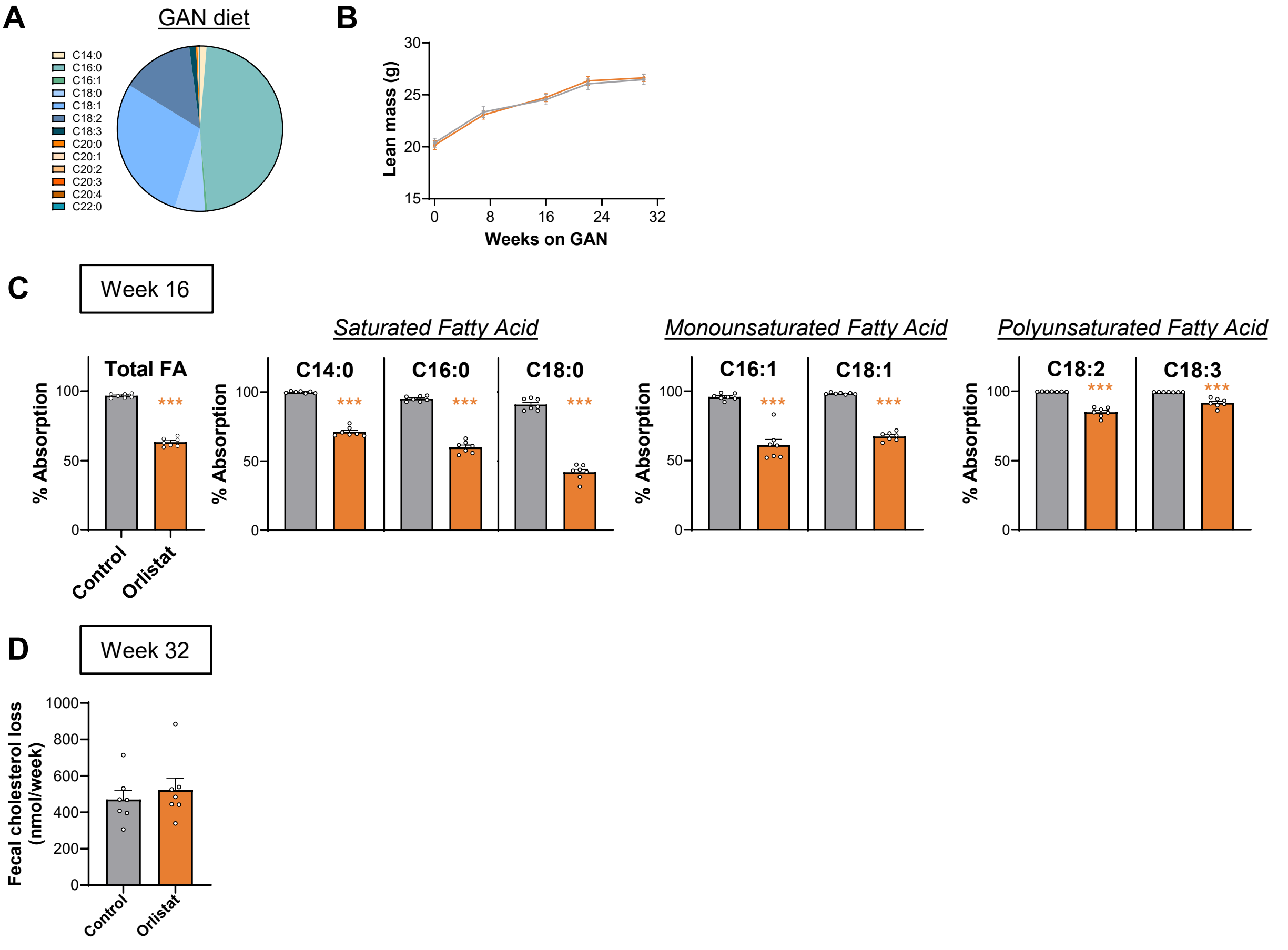

Supplemental Figure 1: Intestinal lipase inhibition with orlistat attenuates GAN diet-induced obesity by promoting fecal fat excretion.

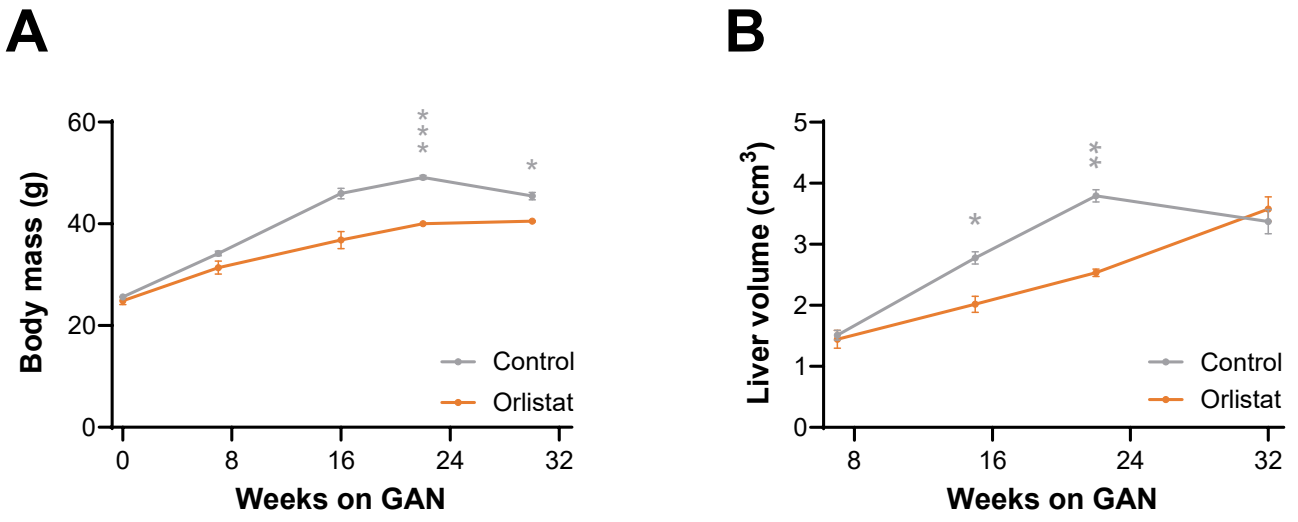

Supplemental Figure 2: Lipase inhibition fails to prevent progression of liver disease on ultrasound imaging.

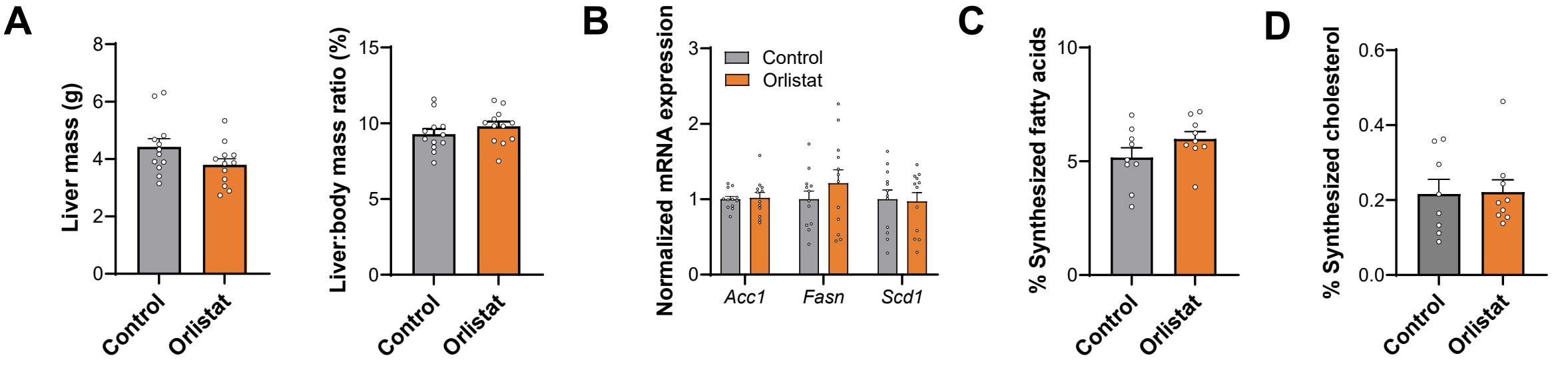

Supplemental Figure 3: Hepatic inflammation persists with lipase inhibition despite reduced weight gain.

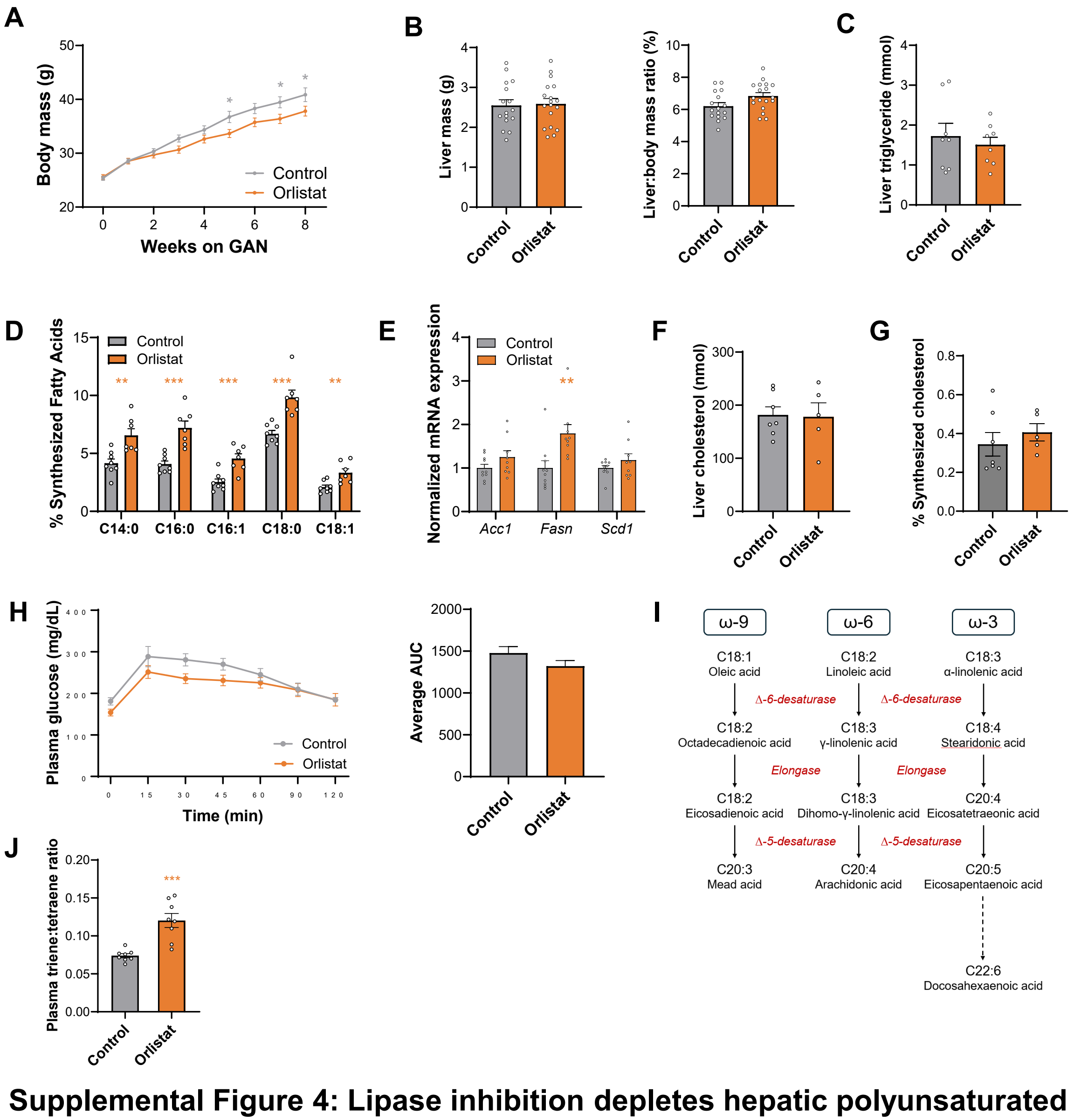

Supplemental Figure 4: Lipase inhibition depletes hepatic polyunsaturated fatty acids and activates *de novo* lipogenesis.

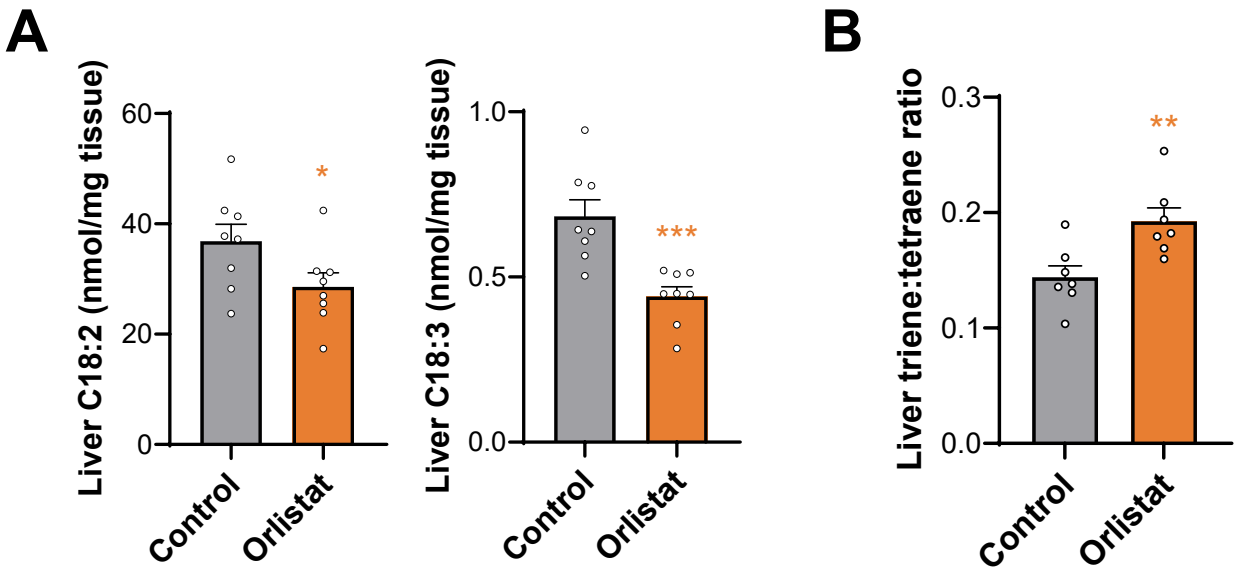

Supplemental Figure 5: Lipase inhibition is associated with persistent hepatic pro-inflammatory signaling.

Supplemental Table 1. Primer sequences used.

Primer ID	Sequence 5'-3'	Purpose
FasnF	GCTGCGGAAACTTCAGGAAAT	*Fasn* qPCR
FasnR	AGAGACGTGTCACTCCTGGACTT	*Fasn* qPCR
AcacaF	TGACAGACTGATCGCAGAGAAAG	*Acaca* qPCR
AcacaR	TGGAGAGCCCCACACACA	*Acaca* qPCR
Scd1F	CCGGAGACCCCTTAGATCGA	*Scd1* qPCR
Scd1R	TAGCCTGTAAAAGATTTCTGC	*Scd1* qPCR
IfngF	TCCTCGCCAGACTCGTTTTC	*Ifng* qPCR
IfngR	GTCTTGGGTCATTGCTGGAAG	*Ifng* qPCR
TnfF	CCGTGTAAAAGCTTAGGGAATGGGTGTTCATCCA	*Tnf* qPCR
TnfR	AGACTCGAGCGGCCGCTCACATTTCTTTTCCAAGC	*Tnf* qPCR
Ccl2F	CATCCACGTGTTGGCTCA	*Ccl2* qPCR
Ccl2R	GATCATCTTGCTGGTGAATGA	*Ccl2* qPCR
Ccl3F	TGTACCATGACACTCTGCAAC	*Ccl3* qPCR
Ccl3R	CAACGATGAATTGGCGTGGAA	*Ccl3* qPCR
NfkbF	CCTGGCCAGTGTAGCAGTCT	*Nfkb* qPCR
NfkbR	AGAGGCTAGGTGCAGACACG	*Nfkb* qPCR
Nlrp3F	CTTGGAGACACAGGACTCAGG	*Nlrp3* qPCR
Nlrp3R	AGGTGAGGCTGCAGTTGTCTA	*Nlrp3* qPCR
Il1bF	TGTAATGAAAGACGGCACACC	*Il1b* qPCR
Il1bR	TCTTCTTTGGGTATTGCTTGG	*Il1b* qPCR
Col1aF	CAGGGTATTGCTGGACAACG	*Col1a* qPCR
Col1aR	GAAGACCAGGGAAGCCTCTT	*Col1a* qPCR
Timp1F	GCAACTCGGACCTGGTCATAA	*Timp1* qPCR
Timp1R	CGGCCCGTGATGAGAAACT	*Timp1* qPCR
Tgfb1F	TGGAGCAACATGTGGAACTC	*Tgfb1* qPCR
Tgfb1R	GTCAGCAGCCGGTTACCA	*Tgfb1* qPCR

Tgfb2F	CAGGAGTGGCTTCACCACAAAG	*Tgfb2* qPCR
Tgfb2R	TGGCATATGTAGAGGTGCCATCA	*Tgfb2* qPCR
TbpF	GGCGGTTTGGCTAGGTTTCT	*Tbp* qPCR
TbpR	ACACCATGAAATAGTGATGCTGG	*Tbp* qPCR

Supplemental Table 2. Antibodies used.

Antibody	Manufacturer	Catalog No.	Host
PDI (1:10,000)	Cell Signaling Technology	3501S	Rabbit (mAb)
ACC (1:10,000)	Cell Signaling Technology	3676S	Rabbit (mAb)
FASN (1:10,000)	Cell Signaling Technology	3180S	Rabbit (mAb)
SCD1 (1:10,000)	Cell Signaling Technology	2794S	Rabbit (mAb)
Anti-Rabbit (1:10,000)	Cytiva	NA934-1ML	Donkey (Polyclonal)
