# Supplementary material for: Intestinal lipase inhibition uncouples diet-induced obesity from MASH by remodeling the hepatic lipidome in mice
Source: J Lipid Res. 2026 Jul 27;67(8):101112. doi: 10.1016/j.jlr.2026.101112 (PMC13521235; doi:10.1016/j.jlr.2026.101112)
Supplement: Supplementary file 3 [file mmc3.docx]

**Supplemental Table 1. Primer sequences used.**

| Primer ID | Sequence 5’-3’ | Purpose |
| --- | --- | --- |
| FasnF | GCTGCGGAAACTTCAGGAAAT | *Fasn* qPCR |
| FasnR | AGAGACGTGTCACTCCTGGACTT | *Fasn* qPCR |
| AcacaF | TGACAGACTGATCGCAGAGAAAG | *Acaca* qPCR |
| AcacaR | TGGAGAGCCCCACACACA | *Acaca* qPCR |
| Scd1F | CCGGAGACCCCTTAGATCGA | *Scd1* qPCR |
| Scd1R | TAGCCTGTAAAAGATTTCTGC | *Scd1* qPCR |
| IfngF | TCCTCGCCAGACTCGTTTTC | *Ifng* qPCR |
| IfngR | GTCTTGGGTCATTGCTGGAAG | *Ifng* qPCR |
| TnfF | CCGTGTAAAAGCTTAGGGAATGGGTGTTCATCCA | *Tnf* qPCR |
| TnfR | AGACTCGAGCGGCCGCTCACATTTCTTTTCCAAGC | *Tnf* qPCR |
| Ccl2F | CATCCACGTGTTGGCTCA | *Ccl2* qPCR |
| Ccl2R | GATCATCTTGCTGGTGAATGA | *Ccl2* qPCR |
| Ccl3F | TGTACCATGACACTCTGCAAC | *Ccl3* qPCR |
| Ccl3R | CAACGATGAATTGGCGTGGAA | *Ccl3* qPCR |
| NfkbF | CCTGGCCAGTGTAGCAGTCT | *Nfkb* qPCR |
| NfkbR | AGAGGCTAGGTGCAGACACG | *Nfkb* qPCR |
| Nlrp3F | CTTGGAGACACAGGACTCAGG | *Nlrp3* qPCR |
| Nlrp3R | AGGTGAGGCTGCAGTTGTCTA | *Nlrp3* qPCR |
| Il1bF | TGTAATGAAAGACGGCACACC | *Il1b* qPCR |
| Il1bR | TCTTCTTTGGGTATTGCTTGG | *Il1b* qPCR |
| Col1aF | CAGGGTATTGCTGGACAACG | *Col1a* qPCR |
| Col1aR | GAAGACCAGGGAAGCCTCTT | *Col1a* qPCR |
| Timp1F | GCAACTCGGACCTGGTCATAA | *Timp1* qPCR |
| Timp1R | CGGCCCGTGATGAGAAACT | *Timp1* qPCR |
| Tgfb1F | TGGAGCAACATGTGGAACTC | *Tgfb1* qPCR |
| Tgfb1R | GTCAGCAGCCGGTTACCA | *Tgfb1* qPCR |
| Tgfb2F | CAGGAGTGGCTTCACCACAAAG | *Tgfb2* qPCR |
| Tgfb2R | TGGCATATGTAGAGGTGCCATCA | *Tgfb2* qPCR |
| TbpF | GGCGGTTTGGCTAGGTTTCT | *Tbp* qPCR |
| TbpR | ACACCATGAAATAGTGATGCTGG | *Tbp* qPCR |

**Supplemental Table 2. Antibodies used.**

| Antibody | Manufacturer | Catalog No. | Host |
| --- | --- | --- | --- |
| PDI  (1:10,000) | Cell Signaling Technology | 3501S | Rabbit (mAb) |
| ACC  (1:10,000) | Cell Signaling Technology | 3676S | Rabbit (mAb) |
| FASN  (1:10,000) | Cell Signaling Technology | 3180S | Rabbit (mAb) |
| SCD1  (1:10,000) | Cell Signaling Technology | 2794S | Rabbit (mAb) |
| Anti-Rabbit  (1:10,000) | Cytiva | NA934-1ML | Donkey (Polyclonal) |
